# The Pool-Boiling-Induced Deposition of Nanoparticles as the Transient Game Changer—A Review

**DOI:** 10.3390/nano12234270

**Published:** 2022-12-01

**Authors:** José Pereira, Ana Moita, António Moreira

**Affiliations:** 1 IN+, Center for Innovation, Technology and Policy Research, Instituto Superior Técnico, Universidade de Lisboa, Av. Rovisco Pais, 1049-001 Lisboa, Portugal; 2 CINAMIL—Centro de Investigação Desenvolvimento e Inovação da Academia Militar, Academia Militar, Instituto Universitário Militar, Rua Gomes Freire, 1169-203 Lisboa, Portugal

**Keywords:** nanofluids, fouling resistance, re-suspension, hydrodynamic instability, thermal resistance, roughness-to-particle size ratio

## Abstract

It is widely known by the scientific community that the suspended nanoparticles of nanofluids can enhance the thermophysical properties of base fluids and maximize pool-boiling heat transfer. However, the nanoparticles may undergo extended boiling times and deposit onto the heating surfaces under pool-boiling conditions, thus altering their intrinsic characteristics such as wettability and roughness over time. The present study reviews the fundamental mechanisms and characteristics of nanoparticle deposition, and its impact on surface roughness and wettability, density of vaporized core points, and thermal resistance, among other factors. Moreover, the effect of the nanoparticle layer in long-term thermal boiling performance parameters such as the heat transfer coefficient and critical heat flux is also discussed. This work attempts to highlight, in a comprehensive manner, the pros and cons of nanoparticle deposition after extended pool-boiling periods, leading the scientific community toward further investigation studies of pool-boiling heat-transfer enhancement using nanofluids. This review also attempts to clarify the inconsistent results of studies on heat transfer parameters using nanofluids.

## 1. Introduction

**Figure 1:**
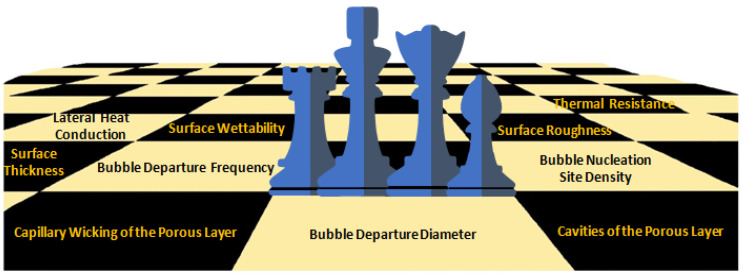
Nanoparticle deposition time-dependent features at play.

The boiling process has been used to dissipate energy in the form of heat generated by macroscale operating equipment. As miniaturized, faster and more powerful electronic devices and systems are developed and implemented on a day-to-day basis, the operating thermal fluids need to be improved to augment the heat removal capability. Pool-boiling heat transfer depends on factors closely linked with the fluid and solid surface properties such as thermal conductivity, surface tension, viscosity, enthalpy, specific heat, and roughness, structure, and homogeneity of the heating surface. It also depends on the hydrodynamic state near the heating surface determined by the bubble departure frequency and diameter and hot/dry spots dynamics [1], among other factors. The suspension of nanoparticles into the base fluid, or in other words using a nanofluid, is one of the most followed routes to enhance the pool-boiling heat-transfer coefficient (HTC) and critical heat flux (CHF) and can modify many of the aforementioned factors and properties. The use of nanoparticles may have considerable influence on the thermophysical properties of the working thermal fluids and on the behavior of the triple solid–liquid–vapor contact line. The alteration of the liquid/vapor and solid surface tensions changes the acting force balance and length of the contact line [2], wettability, and number of active nucleation sites. The focus of the research studies on the field should be to achieve an optimized combination of the base fluid, concentration, shape, and dimensions of the nanoparticles and solid surface substrate to increase, at the same time, the CHF and HTC of the pool-boiling scenarios. Nevertheless, there are still features of vital importance that should be further studied such as the impact and control of the transient boiling-induced nanoparticle deposition [1]. In the course of the nucleate boiling process with nanofluids, the nanoparticles deposit onto the heating surface over time and can modify its properties including the wettability, water angle hysteresis, roughness, capillary wicking [3,4], and nucleation site density. Particularly, the surface roughness after the nanoparticle deposition is influenced by the fraction and intrinsic thermal properties of the nanoparticles, heating surface roughness, and on the thickness and morphology of the nanoparticle deposition layer. Moreover, the active nucleation site’s density has a relevant role in promoting superior boiling heat-transfer performance, and can be controlled by factors such as the surface wettability and roughness, surface superheat value, and thermal properties of the fluid [5]. There is an intricate connection between the number of nucleation sites and the roughness and wettability of the heating surface: as the wettability improves, the likelihood of the liquid filling the cavities increases and, consequently, the number of active nucleation points decreases. In the work carried out by the authors Shoghl et al. [6], after the boiling process, using a alumina nanofluid as operating fluid, the deposited nanoparticle layer decreased the heating surface roughness in the case where the size of the nanoparticles was smaller than the roughness of the heat transfer surface. Moreover, Das et al. [7] reported that the smaller nanoparticles fill the cavities of the surface and reduce its original roughness. Since the nanoparticles agglomerate and deposit, the heating surface roughness decreases when the size of the clusters is smaller than the size of the surface cavities. Moreover, in the study conducted by Chopkar et al. [8], it was reported that after pool-boiling experiments using a 0.005 vol. % zirconia nanofluid, the heating surface roughness decreased. Moreover, the researchers revealed that the surface roughness decreased with the increasing number of successive pool-boiling experiments. Additionally, the authors Narayan et al. [9] employed machine vision to determine the nucleation site density after the completion of pool-boiling experiments with alumina nanofluids. The authors found that the number of active nucleation core points increased when the size of the nanoparticles of 47 nm was smaller than the roughness of the heating surface of 524 nm. In this case, the alumina nanoparticles penetrated into large surface cavities and enlarged the number of sites by splitting one active nucleation point into many points. On the other hand, if the nanoparticle size was similar to the average surface roughness of 48 nm, the number of nucleation sites was found to decrease considerably. Furthermore, the atomic force microscopy (AFM) technique was employed by White et al. [10] to measure the heating surface roughness after multiple pool-boiling tests with deionized water and with a 40 nm zinc oxide nanofluid. The research team observed that the surface roughness increased to 440 nm after seven nanofluid pool-boiling experiments. In addition, the heating surface roughness was reported by Bang and Chang [11] to increase after the nanofluid pool-boiling process if the alumina nanoparticles were larger than the surface roughness. Figure 1 shows the intertwined nanoparticle deposition time-dependent features at play in the pool-boiling heat transfer. Furthermore, the published results in the literature showed that the nanoparticle deposition layer affected the wettability more than the surface roughness [12]. In this direction, Buongiorno et al. [1] conducted pool-boiling experiments with nanofluids on smooth stainless-steel surfaces and found that the contact angle was modified because of the alterations in the solid surface tension provoked by the different chemistry and morphology of the deposited layer of nanoparticles.

It was previously reported in the literature that the CHF is usually enhanced by the alteration of the heat transfer surface characteristics [13,14]. Nevertheless, the nucleate boiling heat transfer of nanofluids is a complex phenomenon that depends on many factors such as the operating conditions [15,16]. Moreover, the experimental results so far published on the boiling heat transfer parameters of the nanofluids are not consistent with each other, even under similar experimental conditions [17]. In addition, the demanding time variation of the boiling heat transfer of nanofluids in which the most representative example is the nanoparticle deposition would be one of the main reasons behind such inconsistency of results [18]. To make matters worse, there is no available systematic experimental information or database regarding the transient nanoparticle deposition and its effects on pool-boiling heat transfer using nanofluids. Hence, further in-depth research works are recommended to achieve a better understanding of the mechanisms responsible for the conflicting trends. The main objective of the current work is to provide, given the circumstances, the most possible complete and accurate experimental and theoretical information about the nanoparticle deposition onto the heat transfer surfaces, in an effort to minimize the severe shortage of understanding of such deposition during pool boiling of nanofluids. Table 1 summarizes some of the main recent experimental works on the boiling-induced nanoparticle deposition and its fundamental effects on the poll boiling heat transfer characteristics.

## 2. Nanoparticle Deposition over Boiling Time

### 2.1. Causing Mechanisms

**Figure 2:**
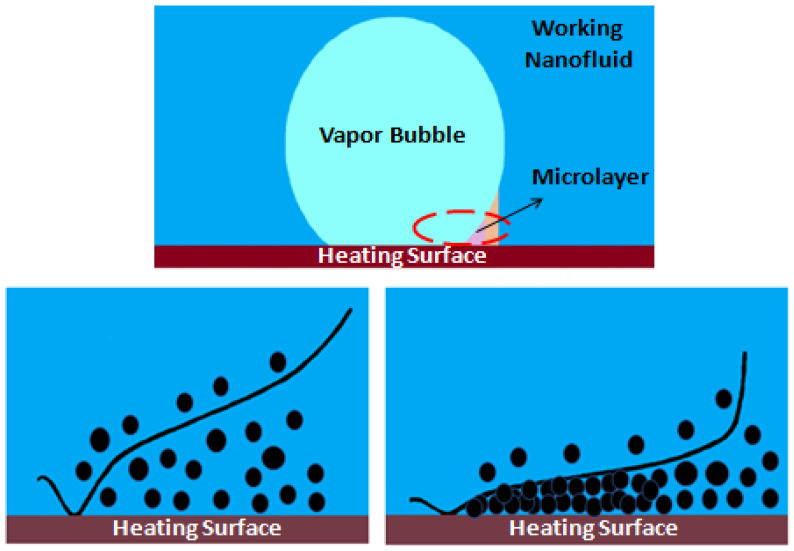
Schematic representation of the nanoparticle deposition in the vapor bubble microlayer. Adapted from [42].

The causing mechanism for the nanoparticle deposition during pool boiling has been pointed out by Modi et al. [38] to be the evaporation in the microlayer beneath the vapor bubbles, where most of the heat and mass transfer is carried out. In this direction, the researchers Li et al. [39] also reported that the microlayer accumulates the nanoparticles that are deposited in the heating surface when the microlayer fully evaporates. The authors found that the microlayer evaporation was the key factor to promote the growing of the bubbles and that the nanoparticle deposition process would continue for as long as the duration of boiling. Figure 2 schematically represents the deposition of the nanoparticles in the microlayer of the vapor bubble. Moreover, Kim et al. reported in another study [40] that the growth of the deposition layer was promoted by the natural convection or by the gravity-driven sedimentation. Furthermore, as the heating surface temperatures increase beyond the operating fluid saturation point, the vapor bubbles are generated at the nucleation sites and start to grow because of the evaporation of the fluid at the contact line, nearby the zone of the bubbles, and inside the liquid microlayer underneath the vapor bubbles [41]. The buoyancy force is responsible for pushing the bubbles upwards until their detachment occurs. In addition, similar mechanisms generate bubbles within the nanofluids during pool boiling. Nevertheless, due to the evaporation rate of the microlayer, the fraction of the nanoparticles in the region around the heating substrate will be enhanced and the distance between the suspended nanoparticles will be shorter. With these conditions, more collisions are likely to happen between the nanoparticles, agglomeration, and sedimentation over the heat transfer surface.

### 2.2. Characteristics of the Deposition Layer

**Figure 3:**
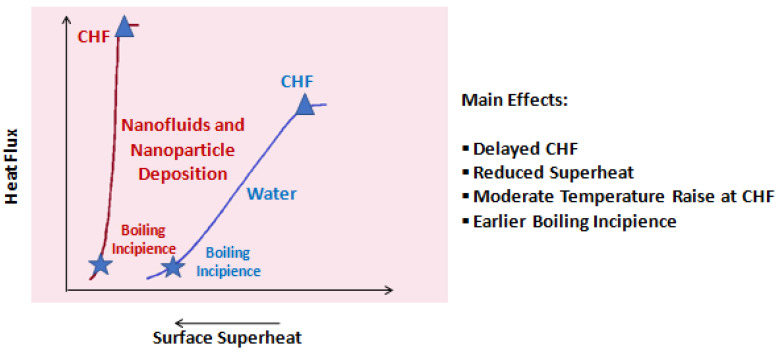
Positive effects of the nanofluids and nanoparticle deposition relative to water. Adapted from [47].

The first characteristic that can be highlighted for the deposited layer of nanoparticles during the pool boiling with nanofluids is the bonding strength with the heating surface. In this sense, the researchers Kwark et al. [43] reported that even after 16 consecutive pool-boiling experiments, the deposition layer kept a noticeable bonding strength with the substrate. Moreover, the deposition layer of nanoparticles has a porous structure over the heating surface [12,17,44] in the nanofluid pool boiling. The thickness of the porous structure and the associated capillarity effect are also relevant characteristics of the deposited nanoparticle layer. The thickness accommodates the extra liquid microlayer and the capillary wicking drives the fluid toward the dry spots at the base of the growing vapor bubbles. Throughout the pool-boiling process, the characteristics of the heating surface covered by the nanoparticle layer are continuously modified as the thickness of the layer tends to increase over time. The authors Kim et al. [45] investigated the evolution of the CHF under pool-boiling conditions on a heating NiCr wire covered with porous layers of nanoparticles. The authors obtained different deposit structures by changing the heat flux during the boiling of 0.01 volume fraction of titanium oxide nanoparticles suspended in water. The surface properties of the testing wires were measured to identify the parameters closely linked with the appreciable CHF increase. The investigation team noted that the heat capacity of the surface of the wires was altered due to the nanoparticle deposition and, hence, the heat capacity was not the main factor in interpreting the observed CHF enhancement. This experimental work may lead to the conclusion that when the CHF occurs on a very small heat transfer surface, such as a thin wire, it is described as being the consequence of the liquid dryout beneath the vapor patch associated with the dramatic increment in the surface superheat value caused by the merging of the vapor bubble rather than the hydrodynamic instability. The researchers stated that the thickness of the surface porous structures holding an extra fluid macrolayer and capillary wicking effect to conduct the working fluid toward the dry regions underneath the vapor bubbles were the key parameters responsible for the CHF increment. The thickness and structure of the deposition layer is affected by the concentration, size, and shape of the nanoparticles, and by the temperature of the heating substrate, rate of evaporation, and finally, induced heat flux. For instance, in the cases when the surface presents a very high temperature and, hence, enhanced evaporation rate, the porous layer becomes thicker and more condensed having larger agglomerations. A higher fraction of nanoparticles dispersed in the base fluid usually conducts to a thicker and more condensed deposition layer. Moreover, the morphology and intrinsic characteristics of the nanoparticles affect the deposition pattern during boiling. Another important characteristic is the roughness of the deposited layer, given that its modification during the boiling process will affect the nucleation site density [15,16,17]. Furthermore, it was already found that not only were the nanoparticles constituents of the deposited layer, since this one also contains solvable salts sedimented in the course of the pool boiling using nanofluids [46] and the influence of these compounds needs further understanding. The formation of a deposition layer during boiling requires engineering to minimize, or even eliminate, the negative consequences on the boiling heat transfer of nanofluids. Nevertheless, there are positive impacts encountered in most cases in the nanofluids and the boiling-induced nanoparticle deposition relative to the conventional thermal fluids such as, for instance, water. Figure 3 presents some of these positive effects.

### 2.3. Fundamental Time-Dependent Features

#### 2.3.1. Fouling Resistance

**Figure 4:**
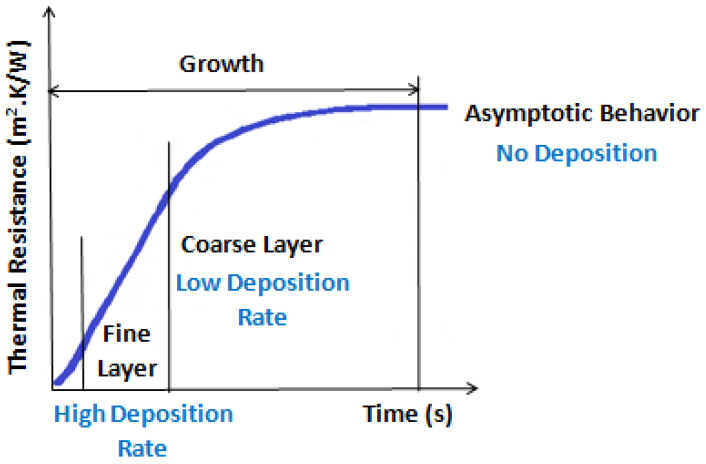
Thermal resistance vs. time and main stages of the particulate fouling. Adapted from [49].

The fouling related with nanofluids is a type of particulate fouling phenomenon. The suspended nanoparticles lose their stability over time and adhere to the heating surface, mainly due to the interactions between the dispersed nanoparticles and the fluid and between the nanoparticles and the heating surface along with temperature gradients within the base fluid [48]. Figure 4 shows the typical time-dependent stages of the particulate fouling.

Moreover, other parameters can be referred that determine the fouling occurrence when using nanofluids such as chemical composition, homogeneity, viscosity, diffusivity, density, interfacial properties, and compatibility of the nanoparticles with each other, the base fluid, and boiling surfaces. Regarding the nanoparticle concentration, an excessive amount of nanoparticles entails sedimentation concerns and, consequently, tends to promote particulate fouling over the surfaces that usually leads to a considerable decrement in the heat transfer performance. It should be stated that another vital concern of the particulate fouling over time is the clogging caused by the clustering of the nanoparticles. The fouling onto a boiling process surface has a considerable negative impact on the working of thermal management units, entailing negative consequences [50,51]. The latter are present in the operational damages caused by the shutdowns provoked by fouling, and in the maintenance costs that arise from cleaning the surfaces and equipment replacement [52]. The heat transfer performance decrement is the most observed effect of fouling [53,54]. This reduction is closely linked with the poor thermal conductivity of the fouling layer, pressure drop increase, clogged equipment, erosion and corrosion of the surfaces, and friction augmentation. To summarize briefly, the boiling fouling by nanoparticle deposition can be addressed through the interplay of the following phenomena [52]:

- Surface particulate deposition is controlled by the interactions between the thermal fluid and nanoparticles and between the heating surface and the nanoparticles.
- Re-suspension of the nanoparticles occurs after the deposition and is determined by the balance between the contact and hydrodynamic forces.
- Agglomeration occurs only in cases where the concentration of the dispersed nanoparticles in the thermal fluid is sufficient to promote consecutive interactions between the surfaces at play. The particle-to-particle collision rate is governed by the hydrodynamic forces associated with the motion of the nanoparticles, whereas the adhesion between the nanoparticles is determined by the short-range interactions between them. In the cases where the adhesion forces have a lower magnitude than the hydrodynamic counterparts, the break-up of aggregates of the nanoparticles may occur.

The hydrodynamic transport of the nanoparticles and attachment onto the heating surface of the nanoparticles are the involved phenomena in the two-step process of the structure of fouling [55,56]. In particulate fouling, the main thermophysical features involved are the motion of the nanoparticles by inertia, diffusion, and thermophoretic forces, linkage of the nanoparticles by the acting Van der Waals forces and superficial charges, and erosion. In pool-boiling scenarios, the fouling of nanoparticles resistance depends on [57]:

- Heat flux: given that with increasing heat flux, the fouling resistance also increases until a maximum value.
- The pH and ionic content of the nanofluids: the attachment and agglomeration of the nanoparticles are provoked by the action of the electric double layer and Van der Waals forces. The repulsion caused by the electric double-layer forces is mainly due to the accumulation of electric charges in the surface of the nanoparticles of hydroxides and metal oxides, and such electric charges are directly correlated with the pH of the suspension and its ionic content.
- Temperature of the surface: it was already demonstrated that the fouling resistance increased more rapidly with temperature at higher temperatures and more slowly at lower temperature values.
- Surface roughness: given that a smoother surface delays the fouling, and it is easier to clean. In contrast, considerable rough surfaces increased the nanoparticle deposition dramatically.
- Size of the nanoparticles: given that the fouling resistance depends on the particle size. However, a well-defined trend is not yet available in the published literature.

The fouling of the copper oxide nanoparticles onto a heat exchanger surface was investigated with a variety of experimental parameters and a correlation accounting with the flow of the nanoparticles was introduced by the authors Nikkhah et al. [58]. The researchers reported that with a larger amount of nanoparticles and increased heat flux, a more considerable surface fouling occurred. Moreover, a reduction in the fouling resistance was found with increasing surface temperature. In particular, the consecutive heating and cooling cycles or the high temperature of the nanofluids tended to promote the impact between the nanoparticles and, consequently, their aggregation trend.

#### 2.3.2. Thermal Resistance

The boiling-induced nanoparticle deposition onto the interface of the solid and liquid phases seriously affects the interfacial thermal resistance (ITR) or Kapitsa resistance. The ITR can be modified by the wettability and morphology of the interface. For instance, the thermal conductance of different wettability interfaces functionalized with a self-assembled monolayer (SAM) has already been determined by the time-domain thermo-reflectance technique [59]. The authors reported that the ITR of a hydrophobic interface was near three-fold greater than that of a hydrophilic interface. Moreover, in the work performed by [5], the layer of carbon nanoparticles deposited onto the solid and liquid phases interface influenced the density of the working fluid, the heat transfer near the interface, and the ITR. The researchers numerically evaluated the ITR with changes in the intermolecular interactions between the fluid and the nanoparticles and those between the fluid and the surface. The researchers arrived at the following conclusions: (i) The nanoparticle layer deposited onto the solid–liquid interface decreased the ITR, which might become lower that the typical ITR value of a smooth heating surface, especially in the cases where the nanoparticles exhibit hydrophilicity. Additionally, it was common in the fullerene and amorphous nanoparticles. (ii) The considerable ITR decrease caused by the deposition layer enhanced the heat transfer from the heating surface to the nanoparticles, and, consequently, augmented the heat transfer of the nanoparticles. The key condition for the decrement in the ITR was the extraction, storing, and transport of the heat from the solid interface by the nanoparticles to the neighboring fluid outside the deposition layer. In addition to the heat flux increment, the temperature difference at the interface of the solid and liquid phases was decreased by the deposition of the nanoparticles. These alterations contributed to the ITR reduction. (iii) The deposit of nanoparticles onto the interface did not induce an appreciably extra thermal resistance as compared with the corresponding fluid layer, independently of the morphology of the nanoparticles. This was caused by the fact that the single layer of the nanoparticles was of such reduced dimensions that it could not be enough for producing the additional thermal resistance at the interface, although the thermal resistance of the used fullerene was high. (iv) The nanoparticle deposition changed the density of the thermal fluid and the temperature gradient at the interface. The ITR decreased with increasing fluid density in the deposition layer. The changes in the fluid density in the interface nearby region and in its temperature gradient by the nanoparticle deposition were two major conditions affecting the ITR.

#### 2.3.3. Three-Phase Contact Line Behavior

During a nanofluid boiling situation, it is usually expected that the dispersed nanoparticles deposit uniformly onto the heating surface by the gravitational effect. Given that the nanofluids are a very dilute type of fluid, the quantity of nanoparticles deposited by gravity can be assumed to be negligible. As such, when the first vapor bubbles nucleate, the heat transfer surface is hydrophobic and the evaporation rate at the solid, liquid, and vapor phases contact line is the main mechanism associated with the growth stage of the initial vapor bubbles. The published transient imaging of the contact line evaporation in the literature has already demonstrated that the contact line of the bubbles presents a radial type of motion across the heating surface [60], provoking the expansion or, alternatively, the contraction of the dry patch. This dry patch can be characterized by a higher temperature area at the center of the active nucleation point. Given that the evaporative heat flux has its maximum value at the solid–liquid–vapor contact line, the suspended nanoparticles will deposit at the contact line as it evaporates and expands. It has been reported that a ring-like deposition pattern was observed at the contact line of a sessile droplet deposit onto a hydrophobic surface due to the delay in the depinning of the contact line. Moreover, it is expected that the radial motion of the contact line will be more constrained with the addition of nanoparticles. As a consequence, the initial deposition happens over only a narrow region under the form of a ring. Furthermore, the deposition at the contact triple line becomes very intense with the boiling time and, thus, modifies the surface wettability that, in turn, aids in the continuous shrinkage of the contact line radius corresponding to the dry patch dimensions. This particular mechanism conducts to the smearing out of the thinned ring to a wider ring structure, which, consequently, improves the wettability of an extended inner region of the nucleation sites. When the rewetting of this region is complete after the detachment of the bubbles, the growth stage of the following bubbles slowly changes from the dynamics of the contact line to the entrapment of the microlayer dynamics. The microlayer thickness augments in the radially outward direction and, consequently, evaporates faster near the center of the nucleation site, leaving behind a small dry patch. Because of the higher evaporation rate at the center of the front of the microlayer, a radially inward capillary flow is generated inside it, which conducts the nanoparticles toward the evaporative front of the microlayer. It should be stated that as the fraction of nanoparticles augments around the evaporative front because of such inward flow, the clustering rate of the nanoparticles raises up to produce larger particles. When the droplet evaporation happens on hydrophilic surfaces, it has been highlighted that the nanoparticles adhere and settle at the contact line region, which is caused by the conjugated action of the shape of the liquid and vapor phases interface and the balance between friction forces and capillarity acting on the nanoparticles. Taking into account these phenomena, it is believed that a similar mechanism is responsible for the settlement of the nanoparticles at the evaporative front of the microlayer considering that the bigger clustered particles come in contact with the evaporative front because of the presence of induced inward capillary flows. The clustering of the nanoparticles at the inner area of the deposition pattern can be easily observed using AFM imaging wherein the sub micro-scaled particles are deposited, and this fact can justify the high density and thickness of the deposit at the central regions of the nucleation point. Moving away from the central regions, although the microlayer possess a relatively large fraction of the working fluid to maintain the suspension of nanoparticles, the thickness of the deposit gradually decreases radially outward since the complete evaporation of the microlayer at these points does not occur because of its increasing thickness. The augmented microlayer thickness renders extra ITR to the heat transfer flow and the microlayer has lower rates of evaporation, which in turn, averts the complete dryout of the microlayer at the radially outward sites. The location of the dry patch can be estimated with the help of heat flux contour plots. If the maximum value of the dry patch radius and the deposited pattern radius is compared, it can be verified that the maximum dry patch radius is significantly smaller than the radius of the deposition pattern. Additionally, it has been found that the maximum dry patch radius is even smaller than the centrally deposited film radius. This fact that in the nanofluid boiling process, the microlayer does not evaporate totally in the radially outward regions and the dispersed nanoparticles settle primarily in such regions because of the gravity and attraction forces between the nanoparticles and surface like Van der Waals and electrostatic forces. Hence, the thickness of the deposit is radially decreased from the center of the nucleation points to their peripheral areas.

#### 2.3.4. Capillary Wicking

Another important feature of the deposited layer of nanoparticles is the capillary wicking behavior. The formation of certain microstructures by the boiling-induced nanoparticle deposition may induce capillary wicking on the heating surface. For instance, in the work performed by Kim et al. [40], the wires covered by the nanoparticles of low fraction nanofluids do not induce a working fluid rise by capillary wicking since the formed microstructures on the heating surface at low concentrations are not sufficient to play the role of microflow pathways. The authors also confirmed that the material of the nanoparticles greatly influences the capillary wicking. In this sense, the oxide titanium nanoparticle-coated wire presented a maximum water capillary rise of 1.2 mm at maximum concentration, which then steeply increased for lower concentrations achieving 5.9 mm at the lowest concentration. Moreover, the alumina nanoparticle covered wire had a maximum water capillary rise value of 0.5 for all the tested concentrations. The fractal micro-scaled structures produced by the deposition of clustered titanium oxide nanoparticles induced fluid suction caused by the capillary wicking effect that increases the CHF of water and turns it considerably higher than that with nanofluids. Nevertheless, the much higher CHF of the heating surface covered by the nanoparticles is deteriorated by the use of nanofluids instead of water, given that the nanoparticles dispersed in the base fluids may clog the microflow pathways conducting the liquid bulk to the heat transfer surface through capillary wicking. Furthermore, the authors Kim and Kim [61] studied the influence of the surface wettability and capillarity of the boiling-induced nanoparticle deposition on the CHF. The authors reported that the deposition of nanoparticles during the boiling process induces capillary wicking on the porous deposition layer, whereby the supplied fluid delays the irreversible growth of dry patches. Moreover, the estimated heat flux gain based on the capillary liquid supply was of the same order of magnitude and consistent with the experimental CHF increase above the maximum CHF value of 1500 kW/m^2^ obtained considering the wettability improvement. The researchers concluded that the appreciable CHF amelioration of nanofluid pool boiling is the result of not only improved surface wettability but also enhanced capillarity caused by the deposition of nanoparticles.

#### 2.3.5. Roughness

**Figure 5:**
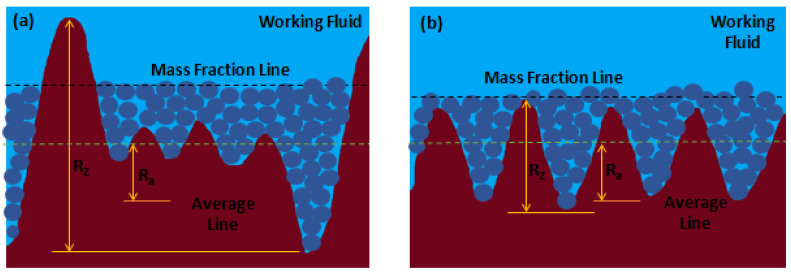
Schematic illustration of the boiling nanoparticle deposition on two different rough surfaces: (**a**) R_a_ and R_z_ with very different values and (**b**) R_a_ and R_z_ with approximate values.

With increasing concentration of the nanofluids, the deposited layer of nanoparticles grows on the heat transfer surface. However, the published results do not always confirm the direct relation between surface roughness and nanofluid concentration. This may be caused by the lack of uniformity of the deposited nanoparticle layer on the heating surface. Such a fact might conduct to higher heat transfer rates with the pool-boiling nanoparticle-deposited surfaces as compared with the ones obtained through the pool boiling of water alone. Nevertheless, it is possible to settle a well-defined correlation between the roughness of the surface and the deposited layer. Moreover, the authors Wen et al. [62] investigated the impact of surface roughness using rough and smooth surfaces. For the smooth surface, the increase in particle deposition on the heating surface led to an increased surface roughness. In the case of the rough surface, no considerable variation in the surface roughness was reported with increasing nanofluid concentrations. The researchers argued that the surface modification was performed by the features associated with the nanoparticles after the boiling process. Furthermore, the surface roughness was found to increase with an increasing wall superheat value for rough surfaces. In addition, the pool-boiling heat transfer of rough surface using nanofluids was around two-fold greater than that of the water alone. A reduction of around 30% in the heat transfer was reported for the pool boiling with nanofluids for a plain surface when compared with the base fluid itself. Although the surface roughness suffers a decrement from 0.167 to 0.099 µm after boiling completion, it achieved a CHF amelioration, which was likely due to alterations in the surface microstructure and topography. Moreover, Ham et al. [3] further investigated the boiling heat transfer of aluminum oxide nanoparticles dispersed in water on heating surfaces with different average roughness. The volume fraction of the nanoparticles ranged between 0 and 0.05 and the average surface roughness was of 177.5 nm and 292.8 nm. The researchers found that when the volume fraction was increased from 0 to 0.05, the CHF increased by 224.8% and 138.5% on the surfaces with roughness of 177.5 nm and 292.8 nm, respectively. The heat transfer capability of the aluminum oxide nanofluid with a 0.05 vol. % of concentration was lower than the one obtained with deionized water at R_a_ = 177.5 nm, but the maximum HTC value increased due to the increase in the CHF. Furthermore, Figure 5 schematically illustrates the boiling-induced nanoparticle deposition on two different rough heating surfaces.

The authors Ji et al. [63] made a qualitative analysis of the boiling-induced nanoparticle deposition on two different rough heating surfaces. The authors stated that it was easier to obtain a uniform deposition layer with a higher R_a_ and lower R_Z_ rough surface in the corresponding 5 (b) case. The mass concentration lines at the same height in the two different cases represent the equality of the concentration. The authors observed too that the heating surface with lower R_a_ and larger R_Z_ is not so easy to be uniformly coated by the nanoparticles. With lower weight fractions of the nanoparticles, it is possible to observe higher peaks outside the surface touching the water. Therefore, the more roughed surface was the one that exhibited improved heat transfer behavior. It can be stated that the number of deposited nanoparticles induces variations in the surface roughness of the heating surface. The continuous growth of the deposited layer produces greater thermal insulation, which leads to the decrement of the CHF. Further experimental studies are needed to establish the optimum value of the deposition layer thickness that maximizes the CHF.

#### 2.3.6. Roughness-to-Particle Size Ratio

The ratio of the nanoparticle dimension to the average roughness of the heating surface can also be linked to the heat transfer behavior. With the use of extended ratios, the deposition of nanoparticles can further enhance the average roughness of the surface or, alternatively, reduce the average roughness. The authors Shoghl et al. [6] found a heat transfer decrement using zinc oxide and alumina nanoparticles dispersed in water. This deterioration was the consequence of the surface roughness decrease as compared with the heat transfer improvement that occurs with carbon nanotubes because of a notorious surface roughness enhancement. The authors reported that any improvement or any degradation of the boiling thermal performance was affected by the nature of the nanoparticles and the relative nanoparticle size ratio to the initial heating surface roughness. In addition, the researchers Narayan et al. [9], Shahmoradi et al. [64], and Wen et al. [65], among others, highlighted that the nucleate boiling enhancement generated by increasing the surface roughness factor was only viable with a well-defined range of size of the nanoparticle. For instance, the authors Narayan et al. [9] introduced the “surface interaction” factor, which represents the ratio of the average surface roughness to the average nanoparticle size, to address the capability of a given nanofluid to improve its nucleate boiling performance. In the cases where the nanoparticles are significantly smaller than the surface roughness features, having a “surface interaction” parameter considerably greater than one, the boiling heat transfer improved appreciably as the smaller particles deposited onto the initial nucleation sites and divided these initial single nucleation points into multiple ones. Nevertheless, in the cases where this interaction parameter had a value near one, the heat transfer was limited as most of the nanoparticles deposited in active nucleation points with approximated relative size and hindered the bubble nucleation. On the other hand, if the referred parameter presented a value considerably smaller than one, the heat transfer decrement was less intense than that verified with the parameter value around one, given that the larger nanoparticles decreased the number of the nucleation points being deactivated. Moreover, the “surface interaction” parameter is also of relevance in interpreting the boiling HTC degradation with augmented concentration of nanoparticles. In this direction, Shahmoradi et al. [64] found a decrement in the HTC for a water-based alumina nanofluid with the “surface interaction” parameter with values lower than one. Such decrement clearly worsened with enhanced nanoparticle volume fraction below 0.1. The researchers explained the worsening deterioration with the additional thermal resistance provided by the deposited layer of nanoparticles. Furthermore, Wen et al. [65] revealed that any augment in the boiling heat transfer was strongly influenced by the relative size of the particles dispersed in the working fluid and the initial dimensions of the superficial elements. Nevertheless, the researchers also highlighted that, since the surface modification by the nanoparticles was a cumulative phenomenon in time, the heat transfer trend was believed to continuously alter with successive pool-boiling experiments with only one heating surface. Moreover, Vafaei [66] reported an increment in the heating surface cavities’ dimensions in the cases where the deposited nanoparticles were larger than the valleys of the surface roughness profile. The reported enhancement promoted the activation of nucleation points and improved the HTC at low heat fluxes. On the other hand, when the settled nanoparticles were smaller than the valleys of the surface roughness profile, the heating surface activation decreased, rendering a less effective boiling performance.

#### 2.3.7. Thickness over Boiling Time

This subtopic has already been discussed throughout the current work, but nevertheless, and concerning the thickness of the deposition layer, it is worth mentioning the experimental work performed by the authors Park et al. [67] who evaluated the effects of the pool-boiling-induced nanoparticle deposition on the CHF improvement. With this purpose, boiling experiments were conducted to evaluate the impact of the thickness of the deposited nanoparticle layer on the CHF using a 0.01 vol. % alumina nanofluid with a Ni–Cr wire as the heating surface. The thickness of the nanoparticle-deposited layer was managed by different boiling times for pre-coating and the CHF evolution curve was acquired in function of the time of the pre-coating process. The authors found that the CHF remain unaltered regardless of the boiling time over a critical pre-coating time, whereas the CHF was dramatically increased during a relatively short pre-coating time. Moreover, the CHF occurrence was moderately decreased after the critical time region. It was also stated that the porosity decreased as the pre-boiling time increased since a denser layer was produced when the boiling time was extended. Moreover, the reduced number of pores affected the CHF decrement after that alteration. This fact denotes that the fluid fraction closely linked with the phase change diminished and this decrease would be promoted when employing a high concentration nanofluid during sufficient pool-boiling time. However, an excessive deposited layer thickness may result in a negative impact on the CHF. Overall, the authors concluded the following:

1. The CHF gradually decreased with long pre-coating times.
2. The CHF improvement may be interpreted by the enhanced surface wettability and hydrodynamic instability modifications with relatively short boiling durations.
3. The slow decrement in the CHF could be interpreted with the porosity decrease in the deposited layer with longer pre-coating durations.

#### 2.3.8. Peripheral Regions of the Nucleation Sites and Deposition Points

**Figure 6:**
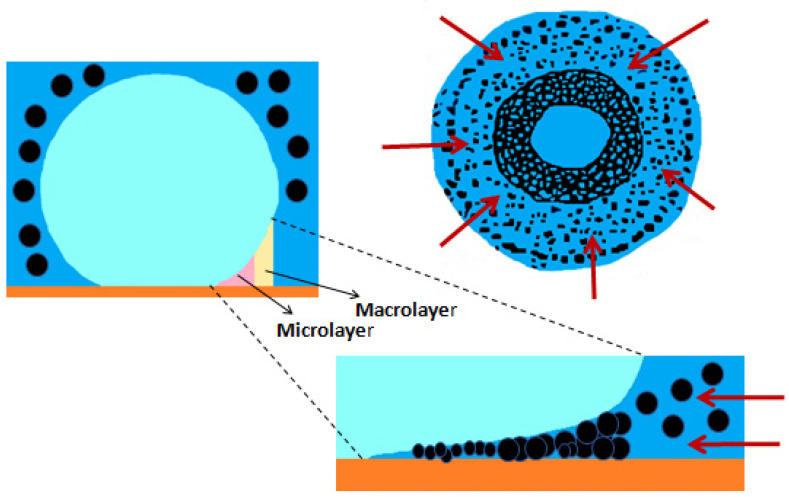
Schematic illustration of the nanoparticle deposition process and top view of the deposited structure showing the size distribution of the nanoparticles.

During the initial part of the growing of the vapor bubbles in the nucleate boiling regime, the viscous effect can be large enough as compared withthe surface tension to hinder the working fluid motion and trap a very thin liquid layer underneath the vapor bubbles beneaththe growing bubbles. This layer is usually designated by a microlayer and has a thickness smaller than ten micrometers and a length up to one millimeter. Its lifetime is only a few milliseconds but it contributes to the major part of the heat transfer enhancement between the heating surface and the bubbles. For longervapor bubble growing times, the formation of a thin liquid layer designated by a macrolayer occurs, wherein the meniscus of the bubbles changes curvature suddenly. In terms of nanoparticle deposition, the particles deposited in the microlayer and macrolayer are usually nano-scaled particles [68]. Another aspect of the boiling-induced nanoparticle deposition process is related to what happens in the peripheral regions of the nucleate boiling points and microparticle deposition points. The periphery of the deposited micro-scaled particles corresponds to the maximum value of peripheral spreading of the area affected thermally by the nucleation sites, beyond which only negligible changes in the temperature are to be observed in the heating surface during bubble growth. This maximum peripheral region corresponds to the macrolayer of the bubbles. Large agglomerated microparticles up to 20 µm have been found in the peripheral region in the form of a ring on the deposited pattern, as it can be seen in Figure 6. Moreover, these micro-scaled particles are considerably larger than the particles deposited in high-density areas near the center of the deposited structure. Considering the associated complexities, it is challenging to interpret whether these large agglomerated microparticles migrated from some region of the liquid bulk to the periphery or, alternatively, whether these particles were formed at the periphery of the nucleation site. In this sense, the work carried out by Kangude and Srivastava [41] made a serious attempt to reveal the most likely phenomena responsible for the presence of the micro-sized particles at the periphery of the active nucleation sites. By AFM imaging observation of the nanoparticle deposition, the researchers confirmed that the nanoparticle deposition during the boiling process led to the formation of a porous deposited structure. They also concluded that the hydrophilicity of the porous deposited layer augmented the capillary flows by offering a chain of hierarchical nano/micro-scaled paths for the fluid to flow. The authors obtained a nanoparticle-deposited structure using the 0.005% nanofluid that was found to exhibit capillary flows.The presence of the capillary flows was ascertained through high-speed camera imaging by observing the motion of the contact line of a sessile droplet deposited onto the nanoparticle deposition layer. It was observed that the droplet achieved static equilibrium first and then the apparent contact line of the sessile droplet moved through the porous layer until it attained a very small contact angle as compared with that of the substrate. However, no such movement of the contact line was verified when the droplet was deposited onto regions of the substrate surface away from the nucleation sites. The authors concluded that the contact line motion confirmed the presence of capillary flows through the deposited porous layer of nanoparticles. Those flows had a strong impact on the microlayer evaporation by continuously replenishing the fluid to the evaporating front of the microlayer. As the microlayer starts evaporating at the central portion of the nucleation site, the capillary pressure forces the fluid to flow toward the contact line of the evaporating front of the microlayer from the periphery through the porous surface of the deposited layer. Furthermore, it should be stated that the thickness and density of the deposit increased from the periphery to the center of the nucleation site. When enough deposition occured, only the working fluid penetrated through the porous layer by capillary forces and the dispersed nanoparticles filtered out toward the peripheral region. The filtered-out nanoparticles agglomerated to form micrometer-sized particles at the periphery over time. Thus, the existence of the micrometer-sized particles in the peripheral region of the nanoparticle deposition points can be reasonably attributed to the presence ofcapillary-assisted radially-inward flows through the pores of the deposition layer during the microlayer evaporation.

#### 2.3.9. Vaporization Core Sites Density

The deterioration of the boiling HTC is caused mainly by the deposition of nanoparticles on the heating surface that enhances the heat transfer resistance. However, the wettability of the surface and the CHF were enhanced. For instance, welding metal foams onto a plain heating surface is a common technique to enhance the heat transfer area. Moreover, this procedure impacts the growth and detachment of vapor bubbles in the course of boiling. Moreover, to enhance the heat transfer capability of the metal foam structure, the researchers Xu et al. [69] studied the influences concerning the size and concentration of AlO and SiC nanoparticles deposited onto a copper foam of 7 mm thickness. The testing pore density was 5 PPI, 60 PPI, 100 PPI, and the corresponding porosity was of 0.9, 0.95, and 0.98. Some preliminary works reported that the boiling induced nanoparticle deposition onto the foamed copper might increase its capillary wicking and number of active vaporization core sites. Moreover, the thermal performance of nanofluids on the gradient hole surface was investigated by Xu and Zhao [70], since adding nanoparticles clogged the voids of the higher 100 PPI density copper foam, and, consequently, the vapor bubbles escape resistance became enhanced and the heat transfer capability degraded. One of the main effects of the heating surface modification is to increase the available heat transfer surface and, hence, enhance the number of available vaporization core sites. Moreover, the density of the nucleated bubbles will become higher in addition to the increasing density of gasification core sites. Therefore, during the pool-boiling process, the nanoparticles deposited onto a bigger heat transfer area, allowing high concentrations of the nanofluid to be employed, rapidly increased the thermal conductivity of the base fluid, and thus the nanoparticles did not deteriorate because of the porous deposition layer. Moreover, the vapor bubbles nucleated on a rough heating surface alter the capillary motion of the vapor bubbles, which are only impacted by the upward action of the buoyancy force.

#### 2.3.10. Hydrodynamic Instability

Another relevant feature of the pool-boiling-induced nanoparticle deposition is the hydrodynamic instability alteration [71]. The vapor bubble dynamics during the pool-boiling process are affected by the deposition of nanoparticles onto the heating surface, which reduces the distance between the vapor bubble departure points. This distance is commonly designated by the Rayleigh–Taylor wavelength instability wavelength or hydrodynamic instability wavelength, which can be easily identified in the film boiling regime. The alteration of this wavelength is linked to the nanoparticle-deposited layer on the heating surface during boiling, given that the deposited layer will alter the spacing between nucleating bubbles (or vapor columns) and, consequently, the Rayleigh–Taylor instability wavelength. In addition, the hydrodynamic instability approach or hydrodynamic fluid-choking limit connects the characteristic wavelength of the hydrodynamic instability with the CHF enhancement of the nanoparticle-deposited porous layer. The hydrodynamic limit theory is based on the Rayleigh–Taylor instability wavelength and was introduced by Zuber [72] for a plain surface and can be broadened to a coated heating surface having capillary limit. Regarding the hydrodynamic limit, the CHF is generally caused by the vapor columns’ instability. The deposited layer can modify the distance between vapor columns on the surface and, consequently, alter the critical instability wavelength. The researchers Liter and Kaviany [73] interpreted the impact of the modulated wavelength on the CHF of a modulated porous layer of nanoparticles (having periodic variations in the layer thickness) using the following expression:

$$Q_{porous}^{\prime \prime }=\frac{\pi }{8}\Delta h_{lv}\sqrt{\frac{\sigma \Delta \rho_{lv}}{\lambda_{m}}}$$

where *λ_m_* is the modulated wavelength or the length scale that defines the vapor escape locations in the working pool-boiling fluid from the porous structure of the heating surface, Δ*h_lv_* is the enthalpy gradient between liquid and vapor phases, Δ*ρ_lv_* is the density gradient between the liquid and vapor phases, and σ represents the fluid surface tension. For a smooth surface, the *λ_m_* parameter is influenced by the balance between the buoyancy force and surface tension, being a function of the thermal characteristics of the working fluid. In the case of a surface coated with a porous layer, the parameter *λ_m_* depends on the vapor escape pathways and, consequently, is a function of the porous structure of the deposition layer. In the experimental work performed by Park et al. [74], the wavelength alteration corresponded to the employed pool-boiling alumina and graphene/graphene oxide nanofluids CHF amelioration trend. As already stated by Liter and Kaviany, the wavelength can be taken as a well-defined geometrical parameter that depends on the surface conditions. As a consequence, the research team found that the change in the wavelength extends the wetting of the heating surface by enabling the working fluid to break through, resulting in the CHF improvement. Nevertheless, the researchers recognized that the recent published CHF enhancement and wavelengths are not consistent with the prediction of the Liter and Kaviany equation. Accordingly with the findings of the aforementioned authors, Park and Bang [71] stated that the onset of the CHF based on the hydrodynamic limit is due to the instability of vapor columns. The nanoparticle porous layer of the deposited nanoparticles during the boiling process could change the critical distance between vapor columns rising from the heating surface and, consequently, alter the critical instability wavelength. Moreover, a similar situation was observed in the droplet formation on the nanoparticle layer, which is closely linked with the detachment of the bubbles from the heating surface. The authors prepared different nanofluids and the used pool-boiling apparatus was designed to enable the direct observation of the Rayleigh–Taylor instability wavelengths. The authors reported that the distance between the bubbles was different for each nanoparticle-coated surface, which revealed a shorter distance between the bubbles than that of the bare heating surface. Moreover, the nanofluids that promoted a higher CHF enhancement exhibited shorter Rayleigh–Taylor instability wavelengths. A short wavelength allows the vapor to prevent the formation of a bulk of vapor by venting the vapor evenly across the heating surface. Furthermore, it was demonstrated that the shorter wavelengths also improved the wettability by allowing the liquid to break through the developing vapor film, which also increases the CHF.

#### 2.3.11. Exfoliation

The deposited layer of nanoparticles on the heating surface may still suffer a peeling action throughout the pool-boiling process. The boiling HTC decreases with increasing fraction of the nanoparticles and the exfoliated area will be increased. This was confirmed by Watanabe et al. [75], who performed experiments to evaluate the force of adhesion of the deposition layer and the effect of its partial detachment on the boiling heat transfer behavior. The researchers reported that the amount of nanoparticles deposited on the heating surface and the force of adhesion of the nanoparticle layer were influenced by the constitutive material of the nanoparticles. With the used nanoparticles, the mass of the deposited nanoparticles could be ordered as titanium oxide > alumina > silica and the force of adhesion was ordered as silica > alumina > titanium oxide. The degradation of the boiling heat transfer was particularly considerable for the titanium oxide with the largest deposited mass. With the peeling of the deposition layer, the settled mass of nanoparticles decreased and the equilibrium contact angle was enhanced. This fact rendered that the degradation of the HTC and increment of the CHF became diminished with the gradually increasing deposition layer detachment. Nevertheless, a larger HTC and a smaller CHF were observed as compared with the bare heating surface for highly exfoliated nanoparticle layers. The authors also stated that the detachment of the deposited layer of nanoparticles happened through a non-homogeneous manner. Moreover, the research team found that the peculiar boiling process at the edge of the remaining deposited layer caused the enhancement of the HTC and the decrement of the CHF.

#### 2.3.12. Re-Suspension of the Nanoparticles

The re-suspension of the deposited nanoparticles of the nanofluids can mitigate or, in some cases, even eliminate the potential deterioration of the nanofluids over time. Indeed, this re-suspension promotes the long-lasting heat transfer amelioration of the nanofluids. The particle re-suspension occurrence is usually observed in nature in, for instance, the re-suspension of the sediments of a riverbed that are carried away by water flow and the re-suspension of sand on the ground, which is blown away by the wind. In particular, the re-suspension of wall-surface-adhering particles by the action of turbulent flows has received great attention over the past few years from researchers. Nevertheless, most of the published scientific articles concerned with nanoparticle suspension comprise single-phase working fluids, and only a few deal with the two-phase fluid condition as it occurs, for example, in boiling. Since the boiling process may induce a very high fluid flow and disturbance of the vapor bubbles, the re-suspension of the nanoparticles deposited onto the heating surface is a very probable phenomenon. If those nanoparticles can be re-suspended, it generally denotes that the time-dependent deterioration of the nanofluids can be relieved without the help of any additional means or methods. This degradation mitigation of the nanofluids over time is a very promising way to achieve long-lasting heat transfer performance. Under pool-boiling scenarios, the movement of the working fluid is induced by convection and movement of the bubbles provoked by buoyancy action. A large part of the existing boiling research is derived from pool-boiling experiments. Consequently, pool boiling seems to be a suitable starting point for the in-depth study of the particle re-suspension behavior that can separate the effect of boiling from the action of the convection-driven flows on the nanoparticle re-suspension. In the work developed by [76], pool-boiling experiments were performed using surfaces with deposited nanoparticles produced by nanofluids. The researchers found the re-suspension ratio of these nanoparticles for different boiling times, densities of the deposition layers, and varying heat fluxes to infer on the impact of the experimental parameters on the re-suspension of the nanoparticles. The authors reported that a certain fraction of the deposited nanoparticles was re-suspended into the liquid bulk and, after that, migrated along with the liquid flow during the boiling process. Since the re-suspension does not take place before boiling, the disturbance suffered by the vapor bubble disturbance in the course of boiling must be an important factor for the re-suspension of the nanoparticles. Besides that, the main difference in the deposition surface between before and after boiling is the porosity of the surface. Hence, it can be stated that the porosity of the deposition layer is the major contributor for the re-suspension of the nanoparticles in the liquid bulk during the boiling process. For a better understanding, it can be noted that when a vapor bubble is in its nucleation state of evolution, the fluid near the bubble will be vaporized because of the superheat. The porous structure of the deposition layer enables the fluid to be vaporized in the layer to be enlarged from the outside of the layer, resulting in a fluid flow from outside toward the inside of the layer. This flow will steadily impinge on the deposited nanoparticles. In the cases where there is no fluid flowing through the deposition layer porous structure, the forces acting on the deposited nanoparticles are gravity, adhesive forces from the interacting neighboring nanoparticles, and brace force. In the cases where the outside-to-inside flow occurs through the pores, the operating fluid will create a dragging force on the nanoparticles around the pore. This force provokes the motion of the nanoparticles that tend to follow the fluid flow. As the generated vapor bubble continues to grow, the drag force increases. At the departure stage, when the vapor bubbles begin their raising from the surface, an extreme disturbing effect at the back region of the departing bubbles occurs. Under these conditions, the drag force overcomes the gravitational effect and forces of adhesion and provokes the detachment of the nanoparticles from the deposition layer, which moves together with the flowing fluid. Given that the fluid flow at the wake of the rising vapor bubbles is also upward driven, the detached nanoparticles will rise upwards together with the liquid flow and are, by this way, re-suspended. Hence, it should be emphasized that two main factors can be assumed for the re-suspension of the nanoparticles: the first is the effect of the fluid flow through the porous structure of the deposition layer during the growth stage of the bubbles and the second is the disturbing effect at the departure of the bubbles. Apart from these factors, many other factors may impact on the nanoparticle re-suspension trend. That is, in the case of the growing and rising process of the vapor bubbles, the nanoparticles dragged from the deposition layer can be re-suspended in the working fluid as a consequence of the departure of the rising bubbles or the breaking-up of the bubbles at the liquid and vapor phases interface. Nevertheless, further experimental and numerical works should be carried out to provide a better understanding of the governing mechanisms of the nanoparticle re-suspension. In the above-mentioned work, the authors plotted the re-suspension ration versus the deposition area density using 20 KW/m^2^, 50 KW/m^2^, 80 KW/m^2^, and 100 KW/m^2^ of heat flux. Moreover, the authors arrived at the following conclusions:

1. During boiling, a fraction of the deposited nanoparticles may be re-suspended within the operating fluid and, after that, migrate along with the fluid flow.
2. The nanopores of the deposition layer have a cardinal part in the re-suspension phenomenon and fluid flow through the pores at the growing stage of the vapor bubbles and the disturbance at the rear zone of the departing bubbles should be taken as the main factors for the re-suspension.
3. The re-suspension ratio increased with increasing heat flux, given that the former increased near 300% when the heat flux was enhanced from 20 to 100 kW/m^2^. Moreover, when low heat fluxes near 20 kW/m^2^ were applied, the re-suspension ratio increased with increasing density of the deposition zone. When there were imposed moderate heat fluxes ranging between 50 kW/m^2^ and 80 kW/m^2^, the re-suspension ratio initially increased and then decreased with increasing deposition area density. When high heat fluxes of around 100 kW/m^2^ were applied, the re-suspension ratio decreased with the density of the deposition area.

**Figure 7:**
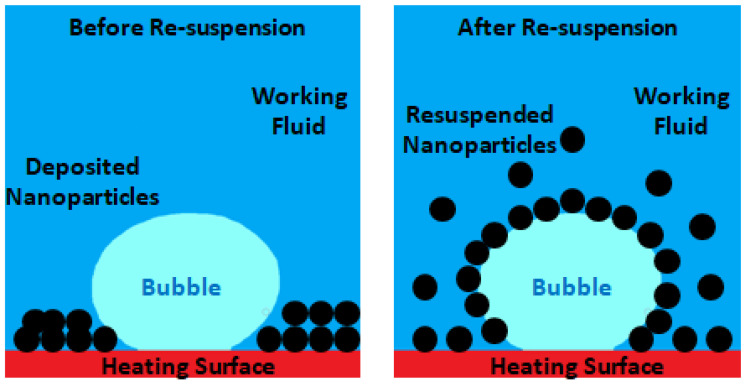
Boiling-induced nanoparticle deposition time-dependent features.

**Figure 8:**
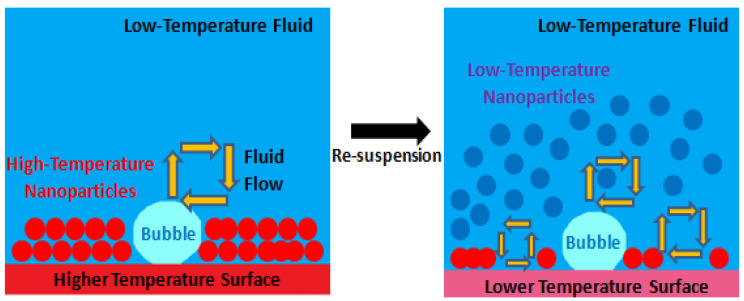
Fluid flow and heat transfer of the nanoparticle re-suspension.

Moreover, Chen et al. [77] studied the re-suspended nanofluid pool boiling under the action of an electric field. The investigation team also discussed the difference in the thermal behavior between the re-suspended nanofluid and the base fluid alone. Figure 7 schematically illustrates the distribution of the deposited nanoparticles before and after the re-suspension phenomenon. Figure 8 shows the fluid flow and heat transfer of the nanoparticle re-suspension.

The authors stated the following conclusions:

1. The nanoparticle re-suspension can be obtained by the action of an electric field and can be demonstrated by the thinned deposition layer of deposited nanoparticles and on the enhanced turbidity of the working fluid.
2. The HTC and CHF enhancement of the re-suspended nanofluid is generated because of the combined effect of the re-suspension and applied electric field. The latter can be enhanced with the voltage augmentation. Nevertheless, there is an optimum value of the concentration of the nanoparticles that maximizes the improving action of the electric field.
3. The mechanisms for the heat transfer improvement of the re-suspended nanofluid under the effect of an external electric field comprise the heat transport between the surface and the fluid promoted by the motion of the nanoparticles, further lowering the surface tension of the liquid and vapor phases interface and the thermal conductivity enhancement of the heat transfer media.

#### 2.3.13. Sintering of the Nanoparticles

The authors Vafei et al. [78] introduced the approach of the nanofluid boiling as a process for the creation of a semiconductor titanium oxide nanoparticle film deposited onto a FTO (F-doped tin oxide) glass conductive substrate. A pool-boiling apparatus was employed to deposit the titanium oxide 20 nmsized nanoparticle nanofluid. The boiling of the nanofluid directly on the FTO glass substrate enables the deposition of the nanoparticles onto its surface. Using the as-deposited films, the crystal growth of the titanium oxide nanoparticles was controlled by altering the temperature, duration, and ramping rate of post-sintering. A densely packed titanium oxide layer was obtained for the as-deposited substrate through the pool-boiling process. For the maximum temperature at 550 °C, the titanium oxide grain sizes became larger and near 50 nm and more round-shaped titanium oxide nanostructures were observed. This work demonstared for the first time how the sintering of titanium oxide nanoparticles proceeds for the nanoporous totanium oxide films. It was observed that the titanium oxide nanoparticles fused with each other and crystal growth happened through the neighboring 2 to 4 nanoparticles at 550 °C. Hence, an extra beneficialfeature of the pool-boiling-induced nanoparticle deposition is that the heating surface will begin to sinter the thin deposited layer. Although this heat treatment is not enough to obtain the required properties, it is enough to increase the stability of the deposited layer. By taking advantage of the stability of the film and the sintering properties of the titanium oxide nanoparticles, a post-sintering treatment after the nanoparticle deposition considerably impacts on the prodictionof a uniform, robust, and dense film. In conclusion, this work demonstrated for the first time that sequential pool-boiling and sintering processes are alternative procedures to create uniform porous titanium oxide layers.

## 3. Effects of the Nanoparticle Deposition on the Boiling Heat Transfer Parameters

### 3.1. Heat Transfer Coefficient

The deposited nanoparticles together with the suspended remaining nanoparticles in the base fluid influence the boiling HTC. The conjunction of the substrate properties such as its base material, the vapor/liquid interface features, and the characteristics of the nanoparticles themselves play a relevant role in the pool-boiling HTC enhancement. This role is performed by the force balance changing and dynamics of the three-phase contact line, bubble growth stage, bubble frequency at departure, and wetting and rewetting. It was predictable that the poor thermal conductivity of the deposition layer increased the conduction thermal resistance and, hence, degraded the HTC. However, the experimental data denied it. In this sense, the experimental work conducted by White et al. [10] showed that the pool-boiling HTC increased with increasing thickness of the deposited layer of titanium oxide nanoparticles. The authors stated that no significant deterioration in the HTC of nanofluids was verified in the course of the deposition. Such a fact may reveal that the impact of the thermal resistance of the deposition layer is not significant when compared with that of the pool-boiling heat transfer by convection, even though the thermal conductivity of the titanium oxide layer is relatively low. Moreover, in the work conducted by Kathiravan et al. [60], the authors used high thermal conductivity nanoparticles of copper and observed that the deposited nanoparticle layer deteriorated the boiling HTC. The already published scientific articles proposed that the influence of the thermal resistance of the deposition layer is not significant and the deterioration of the HTC using nanofluids might be linked with the heating surface wettability and roughness alterations. Additionally, it can be stated that the heat flux directly affects the HTC since at low heat fluxes, the impact of the concentration of the nanoparticles on the pool-boiling HTC is negligible given that at low heat fluxes the larger heating surface cavities are the only ones that are active. Nevertheless, at high heat fluxes, the smaller cavities of heating surface are activated as well but the HTC is reduced with the increasing concentration of the nanoparticles [79]. This effect may be caused by the nanoparticle filling of the smaller surface cavities and by the decrease in the nucleation site’s density. Moreover, in specific studies concerning the impact on the pool-boiling HTC of the surface roughness alterations provoked by the deposition of nanoparticles, it has been proposed that the smaller nanoparticles fill the cavities of relatively rough surfaces and, consequently, decrease the surface roughness and number of active nucleation points, which in turn, demises the pool-boiling HTC and increases the wall superheat value [80,81]. Nevertheless, in the study carried out by Das et al. [7], pool-boiling tests were conducted on a considerably rough heating surface using a 0.005 vol. % zirconia nanofluid. The authors reported that in spite of the heating surface roughness decreasing, the HTC increased. On the other hand, the authors Narayan et al. [9] found that in the case of the average size of alumina nanoparticles being similar to the average surface roughness level, the HTC deteriorated with increasing nanoparticle fraction. In addition, Bang and Chang [11] verified that the pool-boiling HTC became lower when the average surface roughness was inferior to the size of the nanoparticles, and although the surface roughness became higher with the increasing concentration of the nanoparticles, the HTC deteriorated. Furthermore, many researchers reported the increment [79], decrement [41], and lack of reaction [81] of the deposited nanoparticles on the boiling HTC. In conclusion, it should be emphasized that the active nucleation site’s density directly depends on the surface roughness and wettability, and on the average size of the nanoparticles. If the nanoparticles are small compared with the valleys of the surface roughness profile, the active nucleation site density decreases, and if the nanoparticles are not too small when compared with the average roughness of the surface, the nanoparticles will fill and split the surface cavities and the number of available nucleation points will increase. In the cases where the deposited nanoparticles are bigger than the valleys encountered in the surface roughness profile, the nucleation site density might change differently. The flooded cavities are not able to nucleate bubbles. In fact, cavities that are not completely flooded are the ones able to initiate the nucleation of the vapor bubbles and, hence, augment the HTC. The reduction in the surface wettability may avoid flooding its cavities and, in turn, create a higher number of nucleation sites. The authors Forrest et al. [18] studied the influence of the wettability of the heating surface on the HTC using hydrophilic, super hydrophilic, and hydrophobic heating-coated wires. The wires were coated with different surface-treated silica nanoparticles to generate different wettability effects and it must be stated that no visible change was found in the surface roughness after coating, which might indicate that the nanoparticles coated conformably to the micro-scaled surface deformities. The hydrophobic surface was found to possess a higher number of active nucleation sites and higher HTC, the super hydrophilic surface deteriorated the HTC using water, and in the hydrophilic surface, no alteration was observed in the HTC compared with that of the bare heating surface.

### 3.2. Critical Heat Flux

The CHF occurs when a layer of vapor is formed between the thermal fluid and the heat transfer surface. The continuous replacement of this vapor layer by the liquid keeps the heating surface temperature among a safe range. Moreover, the contributing mechanisms for the vapor layer removal from the surface or for the enhancement of the rewetting increased the CHF. Furthermore, the enhancement of the wettability caused by the deposited nanoparticle layer was considered to be a likely reason behind the enhancement of the nanofluid CHF. The effect of the wettability of the surface on the CHF was taken into account in the macrolayer dryout model proposed by Haramura and Katto [82] and in the hot/dry spot theory introduced by the authors Theofanous and Dinh [83]. Nevertheless, further experimental data revealed that all the hydrophobic, hydrophilic, and super hydrophilic surfaces improved the pool-boiling CHF [41]. Considering this fact, it is logical to assume that the wettability might not be the only possible characteristic for the CHF improvement. With the boiling-induced nanoparticle deposition phenomenon, the receding and advancing contact angle and consequent contact angle hysteresis, and the equilibrium contact angle are all subject to change [2]. In the experimental work performed by the researchers Forrest et al. [18], it was confirmed that heating surfaces having small receding contact angles enhanced the CHF. The values encountered for the equilibrium, receding, and advancing contact angles of the hydrophobic heating surface were found to be 140°, 20°, and 160°, respectively. The authors stated that this surface was found to, at the same time, improve the boiling HTC and CHF. Moreover, it should be noted that the lack of homogeneity of the fluids and surface are factors directly influencing the advancing and receding contact angles, and correspondent hysteresis. Furthermore, it is critical to infer on the conditions that increase the nucleate boiling HTC and the CHF of nanofluids simultaneously. Additionally, there are several theories for explaining the boiling mechanisms for the departure from nucleate boiling, which causes a sudden rise in the surface temperature and in the CHF. Such theories include, among others, the hydrodynamic instability, vapor bubble interaction, and the already mentioned macrolayer dryout and hot/dry spot theories. The hydrodynamic instability approach confirms that the hydrodynamic effect associated with the counter current flow of vapor and fluid in the nearby surface region is the fundamental reason for the departure from nucleate boiling. This theory also reclaims that the departure from nucleate boiling happens when the down flow of the fresh fluid to the heating surface is averted by the rising vapor [71]. The macrolayer approach defends that the bubbles are separated from the heating surface by the fluid macrolayer and the departure from nucleate boiling occurs when this macrolayer dries out [84]. The hot/dry spot theory fundamentally deals with the reversibility and irreversibility of hot/dry spots and the departure from nucleate boiling occurs in irreversible hotspots where the rewetting is no longer permitted [85]. In the bubble interaction theory, the departure from nucleate boiling is conducted when the density of the bubbles on the surface is high enough to achieve a complete covering of the surface with a vapor layer, preventing the access of the fluid to the surface [86].

### 3.3. Surface Superheat

The authors Gajghate et al. [35] studied the effect of the ZrO_2_ layer settled during pool-boiling experiments on the heat transfer enhancement. The obtained boiling curves showed the conjugated influence of the nanoparticle layer thickness and surface roughness on the layer superheat value under different heat fluxes. The researchers observed a decrement in the wall superheat value with increasing heat flux and the maximum reduction was verified at 5.8 K with a heat flux of 109.8 kW/m^2^ using a 200 nm ZrO_2_-coated copper substrate having 227 nm of average roughness. The greatest reduction in the surface superheat value was of 31.52% compared with a smooth copper substrate. This effect is caused by the thickness increase in the ZrO_2_ layer deposited onto the copper substrate during pool boiling, which incremented the effective heat transfer surface area and the nucleation core point density. The bubble dynamic at different heat fluxes was also studied showing the nucleation of an isolated vapor bubble on the copper surface at low heat fluxes. With the imposition of high heat fluxes, the bubble diameter was enhanced and lowered the bubble departure frequency. In addition, the authors [42] studied the superheat value of the deposited layer and concluded that it depended mainly on the diameter of the bubbles at the departure stage of evolution and on the boiling time. The layer superheat value was also found to decrease from using the 0.0025% nanofluid to the 0.005% nanofluid. It was observed that the bubble diameter at departure and the boiling time dramatically increased from 0.0025 to 0.005% nanofluid, while keeping almost the same growth time of the nucleated vapor bubbles. With these alterations, the magnitude of the evaporation superheat layer increased steadily with the third power of the diameter of the bubbles at departure and decreased almost linearly with increasing pool-boiling time. Therefore, the conjugated effect of the alteration in the departure diameter of the bubbles and ebullition time led to an increase in the deposition layer superheat value when using the 0.005% nanofluid rather than the 0.0025% nanoparticle concentration nanofluid. The researchers also stated that the HTC associated with each and every heat transfer mechanism was affected by the changes in the bubble dynamics and ITR. These factors are probably determined by the structure in the boiling-induced deposition layer at the active nucleation points. Furthermore, the researchers Hadzic et al. [33] investigated the influence of the nanoparticle dimension and concentration of titanium oxide nanofluids on the pool-boiling heat transport from laser-textured copper substrates. In their work, nanofluid pool boiling was evaluated with 4–8 nm titanium oxide nanoparticles with 0.001 wt. % and 0.1 wt. %. The boiling curve obtained for the 0.001 wt. % nanofluid was stable, while the boiling curve for the 0.1 wt. % nanofluid was shifted toward higher superheat values after the completion of each consecutive pool-boiling experiment. Such a shifting effect may be due to the decrement in the number of nucleation points through the deposition of titanium oxide nanoparticles. Employing the 0.1 wt. % nanofluids, the microcavities on the laser-textured surface were filled with nanoparticles during the initial experiment, which led to a decrease in the number of nucleation sites and an increase in the layer superheat value during the following experiments. With the increase in the number of consecutive experiments, the layer superheat value reached unpractical values and the boiling heat transfer degraded. The boiling experiments were repeated for large (490 nm) titanium oxide nanoparticles. At this time, the authors found with the 0.001 wt. % nanofluid, an appreciable shift in the boiling curve toward lower layer superheat values, while with the 0.1 wt. % nanofluid, the respective boiling curve was observed to be shifted toward higher superheat values. These findings were consistent with those using the smaller nanoparticles. Nevertheless, it was reported that after more than four hours of boiling, the more concentrated nanofluid boiling curve became unstable because of the thick layer settled on the surface that also locally flaked off. An additional study was conducted by mixing 0.05 wt. % small titanium oxide nanoparticles with 0.05 wt. % larger titanium oxide nanoparticles. At this time, the boiling curve was shifted toward a higher layer superheat value in accordance with the behavior observed in the case of the 0.1 wt. % nanofluid having either smaller or larger nanoparticles. It was also found that after the completion of the initial experiment, the layer superheat value was considerably enhanced, which is likely due to the filling of the surface microcavities and channels with the smaller sized nanoparticles that reduced the nucleation points. Moreover, as the latter decreased, the same happened with the boiling HTC. Overall, the authors arrived at the following conclusions:

1. The pool boiling of the nanofluids with higher nanoparticle concentration resulted in a considerable deposition of nanoparticles on the heating surface and a corresponding CHF improvement of up to 2021 kW/m^2^. Nevertheless, were reported very high layer superheat values up to 100 K, which suggested poor practical applicability.
2. The heat transfer decrement of the pool boiling with nanofluids on laser textured surfaces may be explained by the penetration of the nanoparticles into the laser-made grooves and cavities, which decreased the active nucleation site density. Moreover, a thicker deposition resulted in extra thermal resistance, whereas the porosity of the surface assured an appreciable delay in the CHF incipience, and the surface superheat value in turn was dramatically enhanced.

## 4. Main Factors Impacting the Nanoparticle Deposition

### 4.1. Concentration of the Nanoparticles

**Figure 9:**
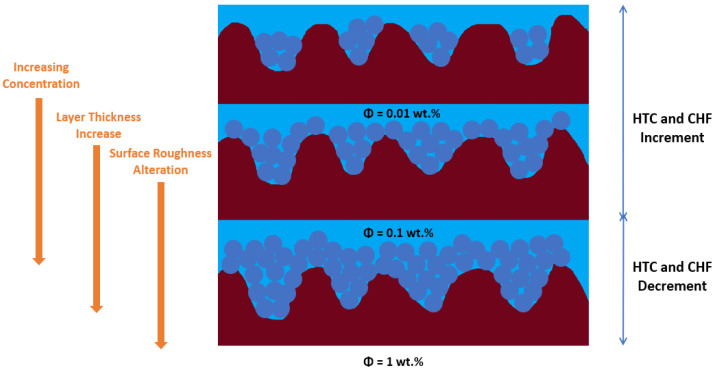
Schematic representation of the pool-boiling-induced nanoparticle deposition for different concentrations and effects on the heat transfer parameters.

The published results showed different characteristics of the natural thermo-convection of the nanofluids according with the concentration of the nanoparticles in the base fluid. One was the boiling heat transfer degradation for, in the majority of cases, volume fractions greater than 0.1. Another was the occurrence of optimum heat transfer rate and coefficient at a certain concentration level, beyond which a decrement was reported. Hence, each and every nanofluid may enhance theboiling heat transfer in an exact volume fraction of included nanoparticles for an exact case depending on the stability of the nanofluid, nature of the base fluid, thermal condition and cavities morphology, and density of the heating surface [87]. As was previouslymentioned throughout the present work, nanoparticle deposition on the pool-boiling heating surface is enhancedwith afraction of the nanoparticles suspended in the base fluid. An excessive nanoparticle deposition may cause the thickening of the deposition layer, which in turn, may lead to an increase in the thermal resistance and, consequently, to the pool-boiling heat-transfer deterioration. This fact was confirmed through the experimental work conducted by the researchers Mukherjee et al. [88] using silica nanofluids having different volume fractions. The authors reported that with lower concentrations of 0.0001 vol. % and 0.001 vol. %, the used nanofluids exhibited limited nanoparticle deposition, inducing a great number of nucleation sites and improving the heat transfer performance. Additionally, when concentrations of 0.01 vol. % and 0.1 vol. % were employed, a greater nanoparticle deposition occurred. The development of a thick deposit further impeded the pool-boiling heat transfer. The results confirmed an increment in the HTC and CHF for 0.0001 vol. % and 0.001 vol. % and a decrement with 0.01 vol. and 0.1 vol. fractions. Furthermore, and according tothe authors Kim et al. [40], the deposition layer produces a continuous modification in the surface morphology that directly influences the boiling heat transfer. Such surface morphology continuous alteration depends strongly on the nanoparticle concentration. In the cases where low nanoparticle volume fractions of 0.0001 and 0.001 were employed, only a slight modification of the heating surface was observed. However, when the concentration was increased, the nanoparticle deposit thickened, and more micro-scaled structures developed on the surface caused by the clustering of the silica nanoparticles. Figure 9 schematically represents the nanoparticle deposition process using different weight fractions of nanofluids. Mukherjee et al. [89] reported that the deposited nanoparticles fill the surface cavities, producing a smoother final surface and also concluded that a grater nanoparticle deposition led to a smoother heating surface. The authors noted that the surface modification is less pronounced at lower concentrations of 0.01 vol. % and 0.1 vol. %, due to the smaller amount of available nanoparticles and the higher stability of the used nanofluids that avert any further settlement of the nanoparticles. Nevertheless, such a tendency changes when the volume fraction is high at 1%. At this concentration, the amount of suspended nanoparticles is considerable and their deposition rate is greater and sufficient to fill up the cavities and alter the texture of the heating surface. Owing to a lesser change inthe surface and improved stability, the nanofluids exhibited enhancements in the HTC and CHF and the opposite trend was found at higher nanoparticle fractions.The effect of nanoparticle concentration in the base fluid was also investigated by Kole and Dey [90]. Two diverse concentrations of zinc oxide nanoparticles having a size of 30–40 nm in ethylene glycol were evaluated. It was observed that, after pool boiling, the nanoparticles were deposited over the heating surface, which prevented the active nucleation sites, and, therefore, the HTC decreased. By measuring the CHF values through the use of a thin copper–nickel alloy, the authors reported a considerable increase in the CHF by increasing the concentration of zinc oxide. The investigation team verified a maximum CHF enhancement of 117% for zinc oxide nanoparticle volume fractions of 2.6%. Moreover, the authors Minakov et al. [36] demonstrated that even at the 0.25 vol. % concentration of nanoparticles, the CHF increased by more than 50% and continued to grow with further increases in the nanoparticle fraction. It was stated that at high concentrations of nanoparticles, the growth rate of CHF slowed down and reached a constant value. Such behavior was due to the stabilization of the deposit size on the heat transfer surface. Moreover, the researchers Ahmed and Hamed [91] studied the pool-boiling heat transfer on smooth copper surfaces using nanofluid and water. Alumina nanoparticles of 40–50 nm were employed to prepare 0.01 vol. %, 0.1 vol. %, and 1 vol. % nanoparticle concentrations, and after performing the boiling experiments, the nanoparticle-coated surfaces were employed for pool-boiling experiments using pure water. The authors found that the concentration of the nanoparticles had great impact on the heat transfer behavior. At lower concentrations, the rate of deposition of the nanoparticles was lower, resulting in a greater improvement of the heat transfer, which can be attributed to the fact that, at low fractions of nanoparticles, the superior thermal conductivity of the nanofluids prevailed over the effect of the nanoparticle deposition onto the heating surface. In addition, the boiling of water on the surface coated with nanoparticles demonstrated that high deposition rates lead to an improvement in the heat transfer, which may be due to the less uniform deposited layer on the surface. Moreover, the authors Coursey and Kim [92] examined the potential of dispersed alumina nanoparticles in water and ethanol. The researchers demonstrated that at low concentrations of alumina, the CHF value remained unchanged, whileat higher nanoparticle concentrations, the CHF value was improved by 37%. In addition, the published results revealed that the carbon nanotube-based nanofluids exhibited appreciably higher thermal features, including thermal conductivity, boiling convective HTC, and CHF compared with the base fluids themselves, as well as other types of nanofluids. These enhanced properties further increased with increasing carbon nanotube concentration and temperature [93].

### 4.2. Size and Shape of the Nanoparticles

The morphology of the nanoparticles has a considerable impact on the thermal conductivity enhancement. For instance, in the experimental work developed by [94], nanofluids were prepared by dispersing titanium oxidenanoparticles in rodshapes measuring10 nm diameter and 40 nm length, and in spherical shapes measuring15 nm diameter in deionized water.The results revealed that the particle size and shape have effects on the thermal conductivity improvement. At 5 vol. %, with the titanium oxiderodsand spheres, the enhancement was found to be around 33% and 30%, respectively, compared with that of the base fluid itself. Moreover, for instance, in the case of ethylene-glycol-based nanofluids [95], the nanoparticle size effect on the thermal conductivity of the nanofluids was also not conclusive as some experimental works reported that the nanofluids with larger-sized nanoparticles exhibitedgreater enhancements in the thermal conductivity than the ones having smaller nanoparticles, whereas others found that the smaller the nanoparticles, the greater the improvement in the thermal conductivity. Hence, the size and shape of the added nanoparticles can also impact on the HTC and CHF. This is related to the fact that different parameters, including post-sintering pore shape, permeability, surface roughness, and thermal conductivity and diffusivity of the deposited layer are influenced by the morphology of the nanoparticles. Moreover, the authors Peng et al. [96] performed an empirical study on the impact of the size of copper nanoparticles on the nucleate pool-boiling heat transfer of a R113/oil thermal fluid. The researchers reported that the maximum increase of 23.8% was achieved for the HTC by reducing the size of the nanoparticles from 80 to 20 nm. Moreover, the smaller copper nanoparticles led to an increased pool-boiling HTC. The impact of the dimensions of the nanoparticles on the boiling behavior was also studied by Hu et al. [97] for a silica nanofluid. The authors observed an increasing tendency of the HTC by decreasing the nanoparticle size from 120 to 84 nm. Furthermore, it can be stated that the nanoparticle size strongly contributes to the amelioration of the pool-boiling CHF, and as the size of nanoparticles increases, better boiling heat transfer performance is achieved for the nanofluids. Additionally, the researchers Minakov et al. [36] established that the CHF using nanofluids depends on the nanoparticle size and that the CHF increased with increasing nanoparticle size. This fact can be explained by assuming that the particle deposition on the heating surface plays the key role in the CHF enhancement. The larger the size of deposited particles, the larger the scale of the final roughness on the surface, which promotes the formation of a deposit thickness enough for enhanced boiling. Shogl et al. [6] showed that the heating surface characteristics depended on the nanoparticles size and surface roughness. Hence, larger nanoparticle sizes improved the boiling performance of the nanofluid. Thus, the carbon nanotubes with water-based nanofluids enhanced the performance of the system and can be considered as the best heat removal method among the examined carbon nanotubes, alumina, and zinc oxide nanofluids, because both surface characteristics and boiling performance were improved with the carbon nanotubes.

### 4.3. Type of Nanoparticles

The authors Shogl et al. [6] performed the evaluation of the boiling performance of zinc oxide, alumina, and carbon nanotube nanofluids under heat fluxes up to 300,000 W/m^2^. The results showed that, using nanoparticles may deteriorate or improve the HTC. To better understand the mechanism of the nanofluid pool boiling, it should be clarified that the decrement or enhancement in the HTC is an intrinsic characteristic of the nanofluids, or is the direct consequence of the heating surface modification, or a combination of both. The deterioration or enhancement depends on the type, size, and surface roughness of the nanoparticles. Inthis experimental work, the zinc oxide and alumina nanofluids formed smoother surfaces and the carbon nanotube CNT formed a rougher surface than the bare one. The overall effect of the zinc oxide/water and alumina/water nanofluids was the worsening of boiling heat transfer, whereas the effect of carbon nanotubes/water nanofluids was the improvement of boiling heat transfer. The type of the nanomaterials can also influence the thermal performance of the boiling systems, given that the different nanoparticle materials result in different thickness and surface relief of the deposits on the heating surface. It was also shown that with decreasing the available heat transfer area of the heating surface along with the boiling-induced nanoparticle deposition, the CHF using nanofluids increased significantly.

### 4.4. Wettability of the Nanoparticles

**Figure 10:**
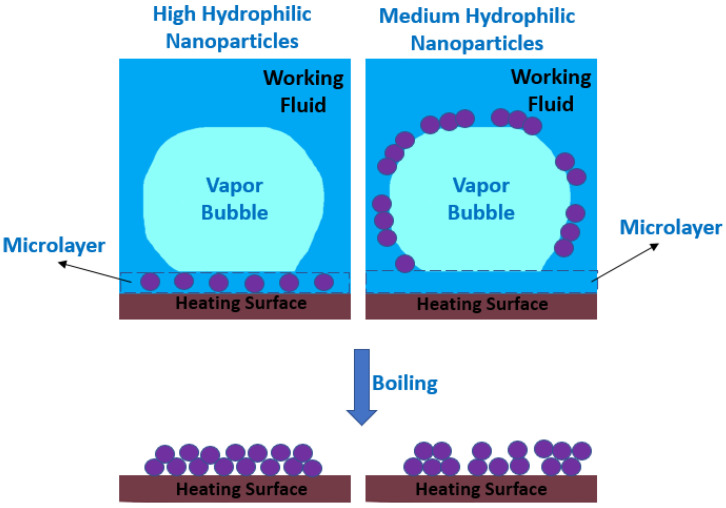
Nanoparticle hydrophilicity effect on the deposited layer morphology.

The nanoparticle wettability can affect the deposited nanoparticle layer morphology. On the one hand, as it can be seen in Figure 10, the suspended nanoparticles with moderate hydrophilicity are adsorbed to the liquid and vapor phases interface avoiding the fluid drainage among the vapor bubbles. This phenomenon hinders the coalescence concern of the bubbles and decreases their diameter at departure, resulting in a nucleate pool-boiling HTC and CHF amelioration. In contrast, the nanoparticles with high hydrophilicity will not adsorb onto the heating surface and, consequently, the bubble coalescence remains unchanged. As illustrated in Figure 10, the wettability of the nanoparticles influences the deposition layer morphology in which the highly hydrophilic layers are relatively smooth having a uniform nanoparticle dispersion onto the heat transfer interface, while the deposited layers produced with moderate to medium hydrophilicity possess higher roughness and more irregularities.

### 4.5. Base Fluid

The differences in the base fluid nature lead to great differences in the thermophysical characteristics of the nanofluid. For instance, if the viscosity of the base fluid is considerable, the viscosity of the nanofluid results higher. Moreover, the disturbance due to the nucleation and departure of the vapor bubbles is the main rationale behind the intense heat transfer. Moreover, the varying viscosity of the nanofluid is critical for the impact on the boiling bubbles because of the differences in the base fluid. It can be found in the published scientific articles that the inclusion of nanoparticles into a base fluid with relatively high viscosity strongly improves the boiling heat transfer. The main features arising from this fact are that the nanoparticles are suspended in a high viscosity fluid, which assures good stability over time. Moreover, the addition of nanoparticles has a negligible impact on the viscosity of the nanofluid that results in only a small alteration in the bubble growth and departure. In summary, the stability of the nanofluid is improved, the nanoparticle deposition is decreased, the number of gasification points together with the surface wettability is enhanced, and, hence, the pool-boiling heat transfer is improved.

### 4.6. Surfactants

The effect of the addition of a surfactant and clustering on the thermal conductivity of titanium oxide and alumina dispersed in water was experimentally studied by the authors [98]. The obtained results showed that the cluster size increased with increasing concentration of nanoparticles, while the thermal conductivity of the nanofluids decreased with increasing cluster size. The CTAB surfactant was effective in improving the dispersion of the nanoparticles and stability of the nanofluids. The added surfactant also contributed to the thermal conductivity of the nanofluids’ improvement. Although the surfactant proved to be a benefit in the stability and thermal conductivity, the effect of the nanoparticle clustering on the same features was found to be negative. The authors Zhou et al. [99] studied the influence of the nanoparticle deposition and interfacial characteristics on the pool boiling using nanofluids and n-butanol as the surfactant over a platinum microwire. The researchers stated that the inclusion of n-butanol altered the liquid/vapor interface properties and it intensified the nanoparticle deposition at low heat fluxes. The obtained results confirmed that the CHF of the nanofluid became enhanced when the n-butanol was added to the nanofluid. The experimental data showed that the hindered bubble growth and increased nanoparticle agglomeration in the fluid wedge region were the reasons behind the degradation of the heat transport deterioration when the amount of the added surfactant was increased. Additionally, it was already proven that the impact of the surfactant on the heat transfer and CHF was greater than the unstable settlement of the nanoparticles onto the heating surface. The deposition profiles were significantly influenced by the n-butanol addition since the low concentration of the self-rewetting fluid produced higher surface tension and, consequently, attracted a greater amount of nanoparticles onto the microwire surface. However, the unstable settlement of the nanoparticles generated a less-uniform deposit as compared with that of the nanofluid alone, which is likely due to the greater disturbance derived from the surfactant addition. In conclusion, when the n-butanol was added to the nanofluid, this surfactant promoted the clustering of the nanoparticles at low heat fluxes. The nanoparticle deposition pattern indicated that this one modified the surface roughness and, thus, accelerated the emergence of the sweeping mechanism of the vapor bubbles. Moreover, it was found that the nanoparticle deposition is more intense with the further addition of a surfactant. The addition of the surfactant will increase the CHF and such an increase is higher for high-nanoparticle concentrations. Moreover, the CHF enhancement ratio decreased when more surfactant was added. In addition, the overall mechanism of the heat transfer enhancement in which the Marangoni flows imposed by the surfactant pushes the nanoparticles to the heating surface to produce the deposition layer and conducts the nanoparticles to penetrate the confined wedge of the vapor bubbles. Furthermore, in the work conducted by the authors Jung et al. [100], the addition of nitric acid as an ionic surfactant promoted the formation of self-assembled layers and structures of nanoparticles on the heat transfer surface. This alternative method builds a more uniform and smoother surface structure, reducing the CHF improvement.

## 5. Limitations and Challenges

Several questions and issues require investigation studies to further understand the main features of the nanoparticle deposition during the boiling of nanofluids. Hence, the following suggestions and challenges are stated for future work:

1. Despite the many published nanofluid-related studies, issues such as long-term stability, erosion, agglomeration, deposition, and maintenance procedures are still obstacles to large-scale commercialization of nanofluids. Hence, studies to extend the actual predictive correlations or innovative numerical simulation tools are highly recommended.
2. The methods for an effective nanoparticle deposition should be reviewed to find a replacement for the deposition rather than the nanofluid boiling; for instance, pre-boiling deposition of nanocoatings on the surface by physical or chemical vapor deposition. However, these depositing techniques require further in-depth investigation studies regarding the optimal thickness of the coatings to observe the delay of the CHF occurrence.
3. Concerning the depositing trend of the nanoparticles in the course of pool boiling, the coating of the surface with particles has been intensively explored to improve the HTC and CHF. In this context, the probable detachment or failure of the layer of particles over boiling time should be further addressed in a laboratory environment. Additionally, the complexity of correlations between the main properties of the heating surface should be addressed to further clarify the mechanisms of amelioration of the heat transfer parameters through the deposition of the nanoparticles.
4. Future experimental works should include the exploration of the possible boiling-induced nanoparticle deposition influencing parameters, including nanoparticle size, shape, type, and substrate material, and base fluid type to the wettability modification and its impact on the pool-boiling heat-transfer characteristics.
5. Regarding the effect of the deposition layer on the boiling surface heat transfer, the thickness of the layer should be optimized to induce the maximum value of latent heat at CHF occurrence.
6. The durability of the nanoparticle deposition layer and its effects on the CHF should be further systematically investigated. The initial studies indicated that the porous nanoparticle deposited layer is affected by the dilatation effect and, under certain conditions, by the formation of a pocket of vapor between the heater and the deposited layer.
7. The particle sorting effect of the boiling-induced nanoparticle deposition requires further in-depth studies. The particle sorting effect has already been observed over the deposited layer of nanoparticles wherein larger micrometer-sized particles, compared with the ones observed close to the center of the deposited layer, have been found in the peripheral regions of the deposited layer. It should be also made clear if this phenomenon is indeed a particle sorting effect or, alternatively, if it is only the result of the boiling-induced capillary wicking flows through the deposition layer toward the center of the nucleation sites.
8. The conjugated effect of the wettability and capillary wicking should be further studied to reveal the heat transfer enhancement for different sizes and fractions of nanoparticles.
9. The bubble dynamics of a single bubble should be further investigated in experimental works and numerical simulations to determine the fraction of the deposited layer and of the suspended nanoparticles that contribute to the pool-boiling heat-transfer amelioration.
10. Efforts should be made to mitigate the initial heating surface differences and, also, to diminish the surface differences occurred during the boiling nanoparticle deposition, given that the modification of the boiling surface geometries is the main factor responsible for the contradictory literature reports on boiling heat transfer with nanofluids. The enhancement or deterioration of boiling heat transfer is dependent upon the relative size between the nanoparticles suspended in the fluid and the heating surface geometry, and respective interactions. Published experimental works already showed that for an initial smooth surface, the deposition of particles increases the surface roughness contributing to the improvement in the nucleate boiling heat transfer, whereas for a starting rough surface, no obvious change in the surface geometry is observed that results in a similar boiling curve.
11. The use of only one heating surface should be avoided, since this procedure will make the quantitative comparison of results more difficult. The experimental evaluation of the nanoparticle concentration effect will also be difficult. The heat transfer surface modification by the deposition of nanoparticles is an intrinsic feature of the use of nanofluids that occurs each time after boiling. Hence, the experimental results are affected by the number and frequency of the usage of the same heating surface.
12. Regarding the effect of the pressure of the pool-boiling system, the growth of the dry patches should be addressed under different pressure levels to elucidate its influence on the heat transfer enhancement from the deposition layer.
13. The possible melting characteristic at some sites on the coated heating surface after confined pool-boiling experiments should be further analyzed. In these cases, the bubble dynamics in the inner confined region and the enhanced residence time of the bubbles on the heating surface underneath could appreciably increase the temperature on the coated layer, melting the nanoparticles through a regressive melting process similar to liquid phase sintering.
14. Further attention must be paid to the possible residual chemical elements dissolved in the working fluid, given that these elements may interact with the deposited nanoparticles and, consequently, modify the morphology and chemical structure of the heating surface and, hence, impair the pool-boiling heat transfer effectiveness. Moreover, this phenomenon tends to take place more under confined boiling in which the hot spots are more sensitive because of the dryout phenomenon.
15. Future studies on the reusability of formerly boiled nanofluids and correspondent stability may be an adequate pathway to better understand the practical implications of the boiling-induced nanoparticle deposition process using nanofluids.
16. To better understand the underlying mechanism of the suspended nanoparticles deposited underneath the vapor bubbles, techniques such as infrared thermography should be further explored to obtain the temperature and heat flux distributions of the active nucleation sites. Additionally, more microscopic measurements of the nucleation site should be carried out with the aid of optical profilometry and AFM to determine the shape of the nanoparticle deposited layer underneath the bubbles.
17. It is suggested that further studies be conducted on repetitive quenching with concentrated nanofluids by monitoring the heating surface wettability alteration and concentration changes after each individual quenching run to better understand the mechanisms of CHF enhancement and achieve a quantitative characterization of the impact of the suspended nanoparticles and its cumulative effect.
18. To better understand how the motion at the microscale of the nanoparticles influences the perturbation suffered by the bubbles in the heating surface, the motion path of the nanoparticles during boiling should be further examined with the aid of a component analyzer and by labeling the nanoparticles. Moreover, it is also advisable to observe and evaluate the generation of bubbles using a high-speed camera.
19. The impact of a multi-component solution on the nucleate pool-boiling heat transfer should be verified through the external condensation of the pool-boiling apparatus, and the relative motion between the different solutions should be observed and discussed. Furthermore, the movement and disturbance of the bubbles should be observed and analyzed by this procedure.
20. It is highly recommended to produce a database that will include the heat transport characteristics together with specific information about the deposited nanoparticle morphology and amount, and dispersion stability with or without the addition of surfactants, in which the enhanced pool-boiling thermal performance of promising nanofluids are prioritized.

## 6. Conclusions

The current work can be characterized as an overview of the transient characteristics of the pool boiling of nanofluids, namely, the nanoparticle deposition advantages and disadvantages during boiling time. The following conclusions should be highlighted:

1. The nanofluids already demonstrated the improvement of the CHF derived from the enhanced wettability of the heating surface after the deposition of the nanoparticles onto the heating surfaces. Nevertheless, the published data concerning the effect of nanofluids on the nucleate boiling HTC are still contradictory. This can be caused by the involvement of intricate concerns such as the nature of the thermal fluid, roughness of the heating surface, and imposed heat flux conjugated with the type, morphology, volume fraction, and preparation and functionalization methods of the nanoparticles. All these factors can significantly alter the thermophysical properties of the nanofluid and certain surface characteristics such as the wettability, surface roughness, number of active nucleation sites, and alterations in the three-phase contact line. Such limiting and complexing issues can strongly restrict the accurate modeling of the nanofluid pool boiling.
2. It was already confirmed that the thickness and density of the deposition layer decreases outward radially when the layer has a spherical shape. Taking into account the morphology and evaporation dynamics of the microlayer, the most verified thickness trend of the deposition layer has been suggested to be caused by the microlayer evaporation phenomenon. Nevertheless, the initiation of the deposition at the active nucleation sites may be caused by the contact line evaporation since such a mechanism is expected to be present in the nanofluid pool boiling.
3. Although the nanofluids have demonstrated great potential in improving boiling heat transfer, there are specific practical concerns that must be considered prior to any usage of the nanofluids in thermal management purposes including the agglomeration, sedimentation, and precipitation of the nanoparticles, equipment and systems clogging, boiling surface erosion, evolution in time of the heat transfer parameters, and inherent overall cost.
4. The nanoparticle deposition onto the heating surface alters its wettability and number of active gasification sites, which affects the heat transfer capability. Moreover, the nanoparticle deposited layer changes the generation of the bubbles and their departure frequency.
5. The morphology of the surface settlement of the various nanoparticles increases the capillary effect, thus enhancing the liquid replenishing after the detachment of the bubbles, which in turn increases the CHF.
6. The development of a closed porous deposit of nanoparticles increases the heat transport resistance of the heating surface and reduces the HTC. Moreover, the deposition causes the CHF to increase more rapidly than the layer superheat value, which results in an increment of the maximum HTC value. It is also common for a significant increase in the thermal conductivity of the base fluid to occur so that the HTC is enhanced. The perturbation of the heating surface caused by the nanoparticles turns the liquid microlayer thinner and enhances the disturbance of the bubbles, resulting in the amelioration of the HTC.
7. The prevalence of larger microparticles in the peripheral regions of the deposition layer has been observed to assume a well-defined circular shape. Nevertheless, the identification of the underlying mechanism still remains unclear, given that it has already been attributed to the eventual nanoparticle sorting or, alternatively, to the pool-boiling capillary wicking through the deposition layer toward the center of the active nucleation sites.
8. It was already observed that the suspension of the nanoparticles in a base fluid with higher viscosity brings benefits to the boiling heat transfer since the deposition of the nanoparticles is smaller; hence, the enhancement of the thermal conductivity of the fluid is more intense than the microscopic motion of the nanoparticles on the surface and the heat transport becomes enhanced.
9. In the cases where the fluid is a multi-component solution, the relative motion between the different composing solutions increases the movement of the nanoparticles due to the different evaporation rates. Therefore, the disturbance of the bubble on the heating surface increases and the boiling heat transfer performance is enhanced.
10. When compared with the thermal conductivity of the heat transfer surface, the deposition of nanoparticles having poor thermal conductivity on the heating surface decreases the heat dissipation and enhances the surface superheat value. Hence, the enhancement of the HTC should be attributed to the thermal conductivity of the nanoparticles and the effect of their movement in the disturbance of the surface vapor bubbles.
11. The different natures of the nanoparticles result in different thermal conductivity and the effects on the pool-boiling heat transfer strongly depend on the deposition pattern. Moreover, only small amounts of the deposited nanoparticles enhance the number of available active nucleation sites and, by this method, the pool-boiling heat transfer capability is improved.

## Figures and Tables

**Table 1:** Main recent experimental works on boiling-induced nanoparticle deposition.

Reference	Authors/Year	Nanoparticles Material	Substrate Material	Effects on Heat Transfer	Other Effects
[19]	Raveshi et al./2013	Aluminum oxide	Copper	An HTC enhancement of up to 64% for the 0.75% vol. nanofluid was verified. Except for the increment of the base fluid properties, the heating surface alteration was the main factor influencing the HTC. Because the surface particle interaction parameter was more than the unity, only the increment of HTC was observed. At low concentration, the deposited layer was very thin, which altered the surface by multiplying the nucleate site and creating active cavities, and finally leading to enhancement of the heat transfer.	The thicker layer, which was recorded at the end of the boiling duration, was caused by the higher concentration of the nanoparticles.
[5]	Sarafraz and Hormozi/2014	Aluminum oxide	Stainless-steel	With increasing the concentration of nanofluids, due to the deposition of nanoparticles on the surface and equal size of nanoparticles and roughness of the heating surface, the average roughness of the surface decreased and, subsequently, the number of nucleation sites reduced which led to an HTC decrease.	An asymptotic behavior was reported for the particulate fouling at the heat transfer area, whereas a rectilinear behavior was found for the free convection region. The thickness and rate of fouling depended on the boiling duration and the fouling resistance because nucleate boiling was higher than those measured in the convection area.
[20]	Tang et al./2014	Aluminum oxide		The aluminum oxide nanoparticles enhanced the heat transfer at concentrations of 0.001 vol. %, 0.01 vol. %, and 0.1 vol. % with the SDBS surfactant. The aluminum oxide nanoparticles deteriorate the heat transfer at 0.1 vol. % without SDBS due to the larger number of deposited nanoparticles. The SDBS deteriorated the heat transfer, and the deterioration was more than the enhancement by the nanoparticles at 0.001 vol. %.When the fractions were between 0.01 vol. % and 0.1 vol. %, the addition of the SDBS improved the heat transfer by the nanoparticles because the SDBS reduced the deposition of nanoparticles.	The impact of the change in the surface angle by the surface deposition of the nanoparticles can be negligible for R141b.
[21]	Manetti et al./2017	Aluminum oxide	Copper	A decrease in the wall superheating up to 32% and 12% for a smooth and rough surfaces, respectively, was verified for the same heat flux in comparison with that of DI water. For low concentration nanofluids subjected to moderate heat flux, appreciable enhancement of the HTC was observed for the smooth and rough surfaces as compared with the DI water. It is argued that this phenomenon is related to the increase in the radius of the cavities due to changes in the morphology of the surface. For the rough surface, the HTC decreased appreciably with increasing heat flux due to the intensification of the nanoparticle deposition rate, and higher thermal resistance by the filling of the cavities of the heating surface with the nanoparticles. The surface modification due to the nanoparticle deposition increased the HTC only for low nanoparticle concentrations and when the particle interaction parameter (SIP) was greater than unity.	A higher nanoparticle deposition rate occurred for heat fluxes greater than 400 kW/m^2^.
[22]	Nunes et al./2020	Aluminum oxide	Copper	The coating layer formed on the heating surface increased the surface wettability; moreover, it provided a barrier to the heat transfer by increasing the thermal resistance on the heating surface, degrading the HTC for unconfined and confined boiling. For the latter, the wettability enhancement promoted a delay in the dryout incipience phenomenon.	The coating process delayed the dryout occurrence under confined conditions due to the influence of the nanostructures on the surface–fluid interaction mechanisms, e.g., the surface wettability, which is a pronounced effect for non-wetting fluids, such as the DI water.
[23]	Xing et al./2016	Carbon	Copper	The multi-walled nanotubes (MWNTs) with CTAB nanofluid presents poor HTC, which decrease with increasing concentration for the deposition of nanoparticles. The deposition of nanoparticles onto the heating surface was not verified using covalent functionalization MWNTs nanofluids. Thus, the covalent functionalization MWNTs nanofluids show a higher HTC than the base fluid, and they increased as the MWNTs concentration increased. The maximum HTC enhancements are 34.2% and 53.4% for MWNTs-COOH and MWNTs-OH nanofluids, respectively.	…..
[24]	Li et al./2020	Copper oxide	Copper	The HTC was improved due to the partial fouling of the nanoparticles which increased the number of nucleation sites on the surface. After 1000 min of operation, the fouling layer changed the surface by decreasing the number of nucleation sites, inducing a thermal resistance to the surface and decreasing the bubble departure time.	…..
[25]	Cao et al./2019	Copper–zinc	Copper	The superheat value on the fully deposited surfaces was around 20 K lower than that on the smooth surface and the fully deposited surface had the highest HTC, around 100% enhancement than the smooth surface. The CHF was not enhanced on the fully deposited surface, but increased by 33% on the channel-pattern-deposited surfaces.	The experimental CHF on the channel pattern deposited surfaces agree well with the predicted model derived from hydrodynamic instability
[26]	Kiyomura et al./2017	Iron oxide	Copper	The coated layer formed on the rough surfaces provided a barrier to the heat transfer and reduced the bubble nucleation, which led to the reduction in the number of microcavities and an increase in the thermal resistance, therefore degrading the HTC. For smooth surfaces, the deposition of nanoparticles tends to increase the nucleation site’s density, increasing the boiling heat transfer. An increment in the HTC occurred only for low nanofluid concentrations, for which the thermal conductivity of the nanofluids was dominant as compared with the thermal resistance of the nanolayer formed on the heating surface.	The C coefficient that correlates to the HTC and the heat flux was used. Different Csf (surface–fluid parameter) and Cs (heating surface parameter) behaviors were found for the surfaces covered with nanoparticles. The Csf and Cs are influenced by the additional thermal resistance resulting from the nanoparticle deposition. The Cs underestimated the effects of wettability and surface roughness for the surfaces covered with nanoparticles
[27]	Stutz et al./2011	Maghemite	Platinum	The coating made of nanoparticles reduced the HTC by introducing a thermal resistance that increased with layer thickness. The CHF enhancement depended on the covering rate of the heating surface by the nanoparticles, and evolved with boiling time. It reached a maximum when the heater was entirely covered with nanoparticles and then decreased slowly when the thickness of the coating increased. The observed increase in the CHF was due to the increase in the heat transfer area when the nanoporous layer was formed.	The effective thermal resistance of the layer appears to decrease substantially during boiling and seems to be coupled to the bubble dynamics. This may indicate that vapor generation occurs inside the layer, which reduces its effective thickness.
[28]	Souza et al./2014	Maghemite	Copper	It was observed that the enhancement of the HTC is higher when the SIP was greater than unity. The HTC for the nanostructured surface with the deposition of nanoparticles of 10 nm diameter, corresponding to SIP = 16, was 55% higher than that for the bare surface. For the nanostructured surface with the deposition of nanoparticles having 80 nm of diameter, corresponding to a SIP equal to 2, the HTC decreased 29%.The HTC increased when the gap decreased, mainly for lower heat fluxes. For a gap length equal to 0.1 mm, a 145% HTC increase at heat fluxes lower than 45 kW/m^2^ with the deposition of 10 nm sized nanoparticles was reported.	….
[29]	Heitich et al./2014	Maghemite	Copper–nickel alloy	Nanostructured surfaces showed higher wettability as a consequence of the greater number of surface defects created by the nanoparticles. Surface defects affect the contact angle and may influence the heat transfer and CHF. The nanostructures have a greater number of these defects due to the small nanoparticle size. The nanostructures led to an increase in the CHF, especially with the maghemite deposition for which the value was around 139% higher than that of the smooth substrate. The CHF increased as the wettability increased. An increase in the CHF was observed as the contact angle decreased. The rough substrate samples showed an enhancement in the HTC of around 19%, while other samples showed an increase in the HTC values for high heat fluxes	The maghemite nanostructured surfaces showed greater porosity and roughness. These samples have a greater nanoparticle layer thickness and, consequently, a higher wettability compared with the molybdenum samples. The rough substrate showed a hydrophobic behavior, while the other samples with nanoparticle deposition showed a hydrophilic behavior. The small particle sizes in the nanostructures greatly promoted the wettability alteration.
[30]	Rostamian and Etesami/2018	Silicon oxide	Copper	Whatever the time of boiling on the heating surface increases, the differences between the boiling curves of nanofluid and deionized water becomes more due to the deposition of nanoparticles. In high concentrations of nanofluid, further deposition of nanoparticles on the surface causes a thickening of the layer made of nanoparticles. This thick layer enhances thermal resistance, so the HTC reduces. However, in low concentrations the surface roughness is less than that in high concentrations and during the nanofluid boiling on the surface, nucleation sites with sedimentation of nanoparticles became smaller in size and the number of nucleation sites increased; therefore, the HTC increased. The main cause for the CHF enhancement in nanofluid was the nanoparticle deposition which increased the surface wettability and CHF was delayed to a higher surface superheat value.	Whenever the concentration and the time of boiling increases, the surface roughness increases, too. Moreover, the increase in surface roughness happens more quickly for higher concentrations (0.01 vol. %).
[31]	Akbari et al./2017	Silver	Copper	The deposition of nanoparticles was efficient in re-entrant inclined coated surfaces: up to 120% CHF increase compared with the smooth surface and up to 30% as compared with the uncoated inclined surface.	The bubbles generated on the coated re-entrant surface were larger in size.
[32]	Kumar et al./2017	Titanium oxide	Nickel–chromium	The CHF was augmented up to a certain value of nanoparticle deposition, beyond which the rate of deposition was intangible. Approximately 80%, 88%, and 93% enhancements in the CHF were found for deposition up to 4 min, 8 min, and 16 min, respectively.	The larger the deposition or boiling time, the lower the contact angle leading to a higher CHF. The rate of deposition of nanoparticles was higher for boiling times up to 8 min and was comparatively lower beyond 8 min and above.
[33]	Hadzic et al./2022	Titanium oxide	Copper	At a low nanoparticle concentration, the influence of nanofluid on boiling performance was minimal, with the HTC and CHF values comparable with those obtained using pure water on both the untreated and laser-textured surfaces. The boiling of a nanofluid with a high nanoparticle concentration resulted in a significant deposition of nanoparticles onto the boiling surface and CHF enhancement up to 2021 kW m^−2^, representing double the value obtained on the untreated reference surface using water. However, very high surface superheat values (up to 100 K) were recorded, suggesting poor practical applicability. The decrease in heat transfer performance due to the boiling of nanofluids on laser textured surfaces can be explained through the deposition of nanoparticles into the laser-induced grooves and microcavities present on the surface, which decreased the number of active nucleation sites.	Thicker nanoparticle deposits resulted in added thermal resistance. While the surface porosity granted a delay in the CHF incipience due to the enhanced liquid replenishment, the surface superheat value was increased.
[34]	Kamel and Lezsovits./2020	Tungsten oxide	Copper	The higher HTC enhancement ratio was 6.7% for a concentration of 0.01% vol. compared with deionized water. The HTC for nanofluids was degraded compared with the deionized water, and the maximum reduction ratio was about 15% for a concentration of 0.05% vol. relative to the baseline case. The reduction in the HTC was attributed to the deposition of tungsten oxide nanoflakes on the heating surface, which led to a decrease in the nucleation site’s density.	…..
[35]	Gajghate et al./2021	Zirconium oxide	Copper	The zirconia nanoparticle coating incremented the heat transfer and HTC. The peak enhancement in the HTC was of 31.52% obtained for 200 nm zirconium-oxide-coating thickness. The HTC increased with increasing coating thickness up to 200 nm, but a further increment in the thickness resulted in the reduction in HTC due to the rise in thermal resistance.	The increase in zirconium oxide nanoparticle concentration from 0.1 to 0.5 vol. % showed an increase in the coating thickness as well as surface roughness. The zirconium oxide layer gave hydrophilicity to the bare copper surface.
[36]	Minakov et al./2017	Silicon, aluminum, iron oxides, and diamond	Nickel–chromium	Even at very small concentrations of nanoparticles, the CHF increased by more than 50% and continued growing with a further increase in the nanoparticle concentration. At high concentrations of nanoparticles, the growth rate of CHF slowed down and reached a constant value. Such behavior can be explained by the stabilization of the deposit size on the heating surface. With increasing boiling time, the CHF increased rapidly and reached a steady-state level. The correlation between CHF and the concentration, particle size and material, and boiling time confirmed the key role of the nanoparticle deposition.	At the same nanoparticle concentration, the desired height of the layer deposited on a smaller heating surface was formed much faster than that deposited on a larger heating surface.
[37]	Sulaiman et al./2016	Titanium oxide, aluminum oxide and silicon oxide	Copper	The aluminum oxide nanofluids enhanced, whereas the silicon oxide nanofluids deteriorated, the heat transfer. The effect of the titanium oxide nanofluid on the heat transfer depended on the nanoparticle concentration. The maximum CHF was found for the most concentrated aluminum oxide nanofluid. Although significant detachment of the nanoparticle layer was found after the CHF measurement for the silicon oxide nanofluids, the value of CHF was not significantly different from those for the titanium oxide and aluminum oxide nanofluids.	Abnormal increase in the wall superheat value was observed for the titanium oxide and silicon oxide nanofluids when the heat flux was sufficiently high. It was considered that this phenomenon was related to the partial detachment of the nanoparticle layer formed on the heated surface since the defects of the nanoparticle layer were always detected when such a temperature rise took place.

## Data Availability

Not applicable.
